# The Effects of Forest Therapy on the Blood Pressure and Salivary Cortisol Levels of Urban Residents: A Meta-Analysis

**DOI:** 10.3390/ijerph20010458

**Published:** 2022-12-27

**Authors:** Quan Qiu, Ling Yang, Mei He, Wen Gao, Harrison Mar, Jiyue Li, Guangyu Wang

**Affiliations:** 1 Guangdong Key Laboratory for Innovative Development and Utilization of Forest Plant Germplasm, College of Forestry and Landscape Architecture, South China Agricultural University, Guangzhou 510642, China; 2 Faculty of Forestry, University of British Columbia, Vancouver, BC V6T 1Z4, Canada; 3 Jiangxi Academy of Forestry, Nanchang 330013, China; 4 Co-Innovation Center for Sustainable Forestry in Southern China, Nanjing Forestry University, Nanjing 210037, China

**Keywords:** forest therapy, blood pressure, salivary cortisol, urban, meta-analysis

## Abstract

Urban residents have a higher risk of hypertension and psychological stress than rural residents. Aside from medical interventions, understanding how to control hypertension and alleviate the stress of urban populations has become a public concern. As an alternative medical measure, forest therapy has shown the effects of normalizing blood pressure (BP) and reducing stress increasingly in recent literature, but this is still inconclusive. In order to systematically verify forest therapy’s effects on the BP and mental stress of urban residents, we conducted meta-analyses to assess the changes in systolic blood pressure (SBP), diastolic blood pressure (DBP), and salivary cortisol concentration (SCC; a stress biomarker) between the forest therapy group and urban control group. We searched 4 online databases, and 21 studies on BP involving 2270 participants and 13 studies on SCC involving 1786 participants were included. Through the meta-analysis, the mean difference and confidence interval (CI) estimates indicated that forest therapy significantly reduced participants’ SBP −3.44 (95% CI −5.74, −1.14), DBP −3.07 (95% CI −5.59, −0.54), and SCC −0.07 (95% CI −0.10, −0.04), as compared with urban control. Yet, there was substantial heterogeneity (*I*^2^ = 72.87–88.59%) among these studies. We also found that each prediction interval (PI) for SBP (95% PI −13.30, 6.42), DBP (95% PI −15.54, 9.41), and SCC (95% PI −0.18, 0.05) were all much wider than the CIs and contained values above 0. This means that forest therapy may reduce SBP, DBP, and SCC on average, but may not exclude adverse results. Meta-regression analyses showed that participants’ age and baseline SBP levels determined the heterogeneity among SBP studies. After forest therapy, older participants and those with higher baseline SBP levels had greater SBP-lowering effects. Among DBP studies, the primary source of heterogeneity was participants’ baseline DBP levels; participants with higher baseline DBP levels had greater DBP reduction. In subgroup analyses, we discovered that longer-term forest therapy programs (≥20 min) resulted in a greater reduction in BP and SCC than shorter-term forest therapy programs (<20 min). Additionally, seated viewing, walking, or multi-session programs in forests were observed to have similar effects on reducing BP and SCC. Despite extensive analyses, the study did not identify any sources of heterogeneity among forest therapy programs for lowering SCC levels. Overall, we conclude that forest therapy programs have beneficial therapeutic effects on urban residents’ physio-psychological health through lowering BP and relieving stress (reducing SCC). This finding provides solid evidence of the contribution of forest therapy to urban residents’ health and wellbeing.

## 1. Introduction

Health risks associated with urban environments exist (such as air pollution, occupational hazards, and traffic hazards), thus posing a major health challenge to urban residents. As a result of multiple stressors (such as odor, noise, light, and vibration) that urban residents face when living in built environments, mental health problems are more prevalent in urban areas than in rural areas [1,2]. It has become a matter of public concern to learn how to alleviate the psychosocial stress of urban populations, aside from medical intervention.

Worldwide, hypertension has been identified as a substantial modifiable risk factor for cardiovascular disease and premature death, with approximately one-third of adults suffering from hypertension [3,4]. The World Health Organization (WHO) estimates that 1.4 billion people around the world suffer from hypertension, whereas only 14% have it under control [5]. As urbanization increases, the prevalence of cardiovascular risk factors will also increase, along with profound changes in physical work, dietary habits, and mental stress occurring [6]. There is increasing evidence that urban populations are more likely to suffer from hypertension than rural populations in South Africa [7], Cameroon [8], and Asia [9,10]. An explanation may be that noise and pollution in urban areas contribute to higher blood pressure (BP) levels [11,12,13]. Notably, a high prevalence of hypertension exists in developing countries, particularly in urban areas, and there are few public awareness and control programs or cost-effective treatment options. For urban residents, especially those diagnosed with hypertension, it is imperative to control their BP through pharmacological treatment. As recommended by WHO, individuals with a confirmed diagnosis of hypertension and a systolic blood pressure (SBP) of ≥140 mmHg or a diastolic blood pressure (DBP) of ≥90 mmHg should initiate pharmacological antihypertensive therapy. In addition, as with psychological stress relief, the public is interested in alternatives or adjuncts to medication to control hypertension.

Although there is some uncertainty regarding whether BP is higher in rural or urban populations [14], several studies have shown that rural residents generally have less severe hypertension [15]. Possibly, this is because they have greater opportunities to interact with natural environments, particularly forests [16]. As stated by Shosha [17], numerous studies support the comprehensive health benefits of the exposure to nature and green environments on human systems. There is significant evidence indicating that forest-based interventions can reduce hypertension, stress, and mental disorders, according to systematic reviews recently presented by Rajoo et al. [18] and Stier-Jarmer et al. [19]. Forest bathing (Shinrin-yoku) was first proposed in Japan as an essential part of a healthy lifestyle by politicians and medical professionals as early as the 1980s. As a relatively new concept, forest therapy has its roots in the Japanese practice of forest bathing and describes a consciously experienced, relaxing stay in the forest [17,20]. The concept is to use all five senses (sight, smell, taste, hearing, and touch) mindfully to immerse oneself in nature. Recently, an umbrella review presented by Antonelli et al. [21] concludes that forest bathing is most strongly supported as a complementary practice for promoting psychological well-being, whereas larger investigations are needed to establish its clinical utility for improving organic diseases. In general, increasing studies have shown that forest therapy or natural exposure programs reduce blood pressure and relieve stress. Yet, they are still controversial and are not universally accepted by the public.

Cortisol is a stress hormone produced by the adrenal glands and can serve as a sensitive and reliable stress biomarker [22,23]. It is becoming increasingly popular with medical professionals to test salivary cortisol levels rather than to test blood or urine cortisol levels because it could potentially cause less stress and can be performed at home using a fully automated nonisotopic assay [24]. The salivary cortisol concentration (SCC) is commonly measured to assess stress levels. Clearly, urban environments can be very stressful to a person’s psychological well-being. Several studies have also confirmed that people exposed to urban environments tend to have higher levels of SCC than those exposed to forest environments [25,26].

Meta-analysis is a statistical method for synthesizing quantitative results from studies with effect estimates and variances [27,28], and it increases the probability of identifying clinically important effects as statistically significant [29]. In previous studies, many scholars have conducted meta-analyses to verify the effects of forest therapy on blood pressure and mental health. For example, Kotera et al. [30] performed a meta-analysis to investigate the effects of forest bathing and nature therapy on mental health, Antonelli et al. [21] conducted a meta-analysis to estimate the effects of forest bathing on SCC, and Ideno et al. [31] used a meta-analysis to assess forest bathing’s impacts on SBP and DBP. These studies provide great help to understand the effects of forest therapy on BP and SCC, but the results are not all identical. Further research is needed to clarify this issue.

To assess the effects of forest therapy on BP and SCC in urban residents, this study used meta-analysis to estimate the change in SBP, DBP, and SCC outcomes between the forest therapy group and urban control group. The following research questions are addressed: (1) do increasing studies further support the observation that forest therapy lowers BP (SBP and DBP) and relieves stress (SCC) in urban residents; (2) is there substantial heterogeneity among studies examining forest therapy’s effects on SBP, DBP, and SCC outcomes; (3) what are the primary sources of heterogeneity among the studies? The purpose of this study is to provide evidence regarding the health benefits associated with forest therapy on urban residents and to propose suggestions to improve the design of forest therapy programs.

## 2. Materials and Methods

### 2.1. Search Strategy

Our systematic review and meta-analysis were conducted in accordance with the Preferred Reporting Items for Systematic Reviews and Meta-Analyses (PRISMA) guidelines [32,33], as shown in Table S1 (Supplementary Materials). Studies of forest therapy effects on BP and SC (published between 1 January 2000 and 31 December 2021) are both indexed in four databases, including Web of Science Core Collection (WoSCC), MEDLINE, PubMed, and China National Knowledge Infrastructure (CNKI). When searching for studies of forest therapy effects on BP, search terms were associated with forest therapy (forest*), urban environment (*urban or city*), and BP (“blood pressure”), and when searching for forest therapy effects on SC, search terms were associated with forest therapy (forest*), urban environment (*urban or city*), and SC (“salivary cortisol”). The detailed search strategy was shown in Table S2.

### 2.2. Study Selection

Using Mendeley software 2. 77. 0 (URL: https://www.mendeley.com/, accessed on 2 March 2022) as a reference management tool, records were downloaded and merged from each database. Titles, abstracts, and full text were screened independently by three reviewers (Q.Q., W.G., and M.H.) to assess eligibility until consensus was reached. The inclusion criteria for this meta-analysis were summarized according to the participants, intervention, comparison, outcomes, and study design (PICOS) format (Table 1). Here, participants are not limited by physical health, in order to observe the therapeutic effects of forests on high blood pressure and mental stress. According to the definition of forest therapy, we decided to collect studies regarding all types of forest therapy activities, such as seated viewing, walking, forest bathing, or multi-session programs. Furthermore, since the objective was to compare the differences between forest and urban environments, studies involving viewing images, videos, or virtual reality simulations of forests were excluded, as well as studies without urban control. In terms of outcomes, only studies that measured the participants’ BP (SBP and/or DBP) or SCC after forest therapy were included. Randomized controlled trials (RCT) are recognized as the gold standard research design for evaluating the effectiveness of healthcare interventions [34]. Indeed, as Antonelli et al. [23] reported, only studies designed using RCT were collected for meta-analysis on the effects of forest bathing on cortisol levels as a stress biomarker. Whereas, in Yao’s study [35], studies designed using RCT and non-randomized controlled trials (Non-RCT) were both collected for meta-analysis of the effect of exposure to the natural environment on stress reduction. Similarly, to collect more studies for meta-analysis, we decided to include all studies designed using RCT (including parallel RCT and crossover RCT) or Non-RCT in this study.

### 2.3. Data Extraction

The following information was extracted from each included study using a standardized form: author’s name, publication year, study location, design, participant’s characteristics, forest therapy procedure (sessions and duration) and control, outcomes, and outcome measures. The data were extracted and incorporated into a coding frame using Microsoft Excel, then synthesized and tabulated. Typically, outcome data about mean, standard deviation (SD), and the number of participants were obtained directly from the included studies, but some estimates and/or calculations were required. If the mean and/or the SD values were not provided in the published articles, we estimated these data from the figures using Webplotdigitizer (Version 4.5; Automeris Software, San Francisco, CA, USA; URL: https://apps.automeris.io/wpd/, accessed on 5 April 2022). If it was desirable to combine two or more reported subgroups into a single group, the mean and SD values were calculated using the standard formulas from the Cochrane Handbook [36]. Here, the SBP and DBP values are displayed as mmHg and the SCC values are shown as μg/dL. In some cases, the SCC values were converted from nmol/L to μg/dL. All data extraction was performed by the first author (Q.Q.). Data extraction forms were critically checked by a third researcher (M.H.) who is certified as a Forest Therapy Guide by the Association of Nature Forest Therapy (ANFT), and disputes were resolved through discussion until a consensus was reached.

### 2.4. Quality Assessment

Non-randomized studies were assessed using the Risk of Bias in Non-Randomized Studies of Interventions (ROBINS-I) tool [37] based on seven criteria: confounding, selection of participants, classification of interventions, deviation from intended interventions, missing data, measurement of outcomes, and selection of reported results. We assessed the included parallel RCT studies using a revised Cochrane Risk of Bias tool for randomized trials (RoB 2) [38]. This tool provides an overall risk of bias for randomized trials, based on scores across five different domains: randomization process, deviations from intended intervention, missing outcome data, measurement of the outcome, and selection of the reported results. We used *robvis* (visualization tool) to create risk-of-bias plots for RoB 2 (for parallel RCT studies) and ROBINS-I assessments [39]. For crossover RCT studies, an Excel tool to implement RoB 2 (URL: https://www.riskofbias.info/welcome/rob-2-0-tool/rob-2-for-crossover-trials, accessed on 15 May 2022) was used to assess their risk of bias and visualize the results. As an addition to the five domains covered in RoB 2, period and carryover effects in a crossover trial were added to the overall risk of bias assessment in this tool. Three independent researchers (Q.Q., M.H. and J.L.) assessed all studies until consensus was reached.

### 2.5. Meta-Analysis

All meta-analyses were conducted using a random effects model (DerSimonian-Laird method), which is considered a more conservative approach suitable for cases of high heterogeneity [40]. The mean difference (MD) between forest therapy and urban control was estimated to explore the effects of forest therapy on urban population. Forest plots with 95% confidence intervals are used to present all results. Heterogeneity was evaluated using *I*-squared statistics (*I*^2^) [41], however, its interpretation has not been clearly defined. Based on a rough guide to explanatory heterogeneity in the Cochrane handbook [36] and Yao’s study [35], values of *I*^2^ < 30% represent no heterogeneity, values of 30–60% reflect moderate heterogeneity, and values of >60% represent substantial heterogeneity. Tau-squared statistics (τ^2^) were also used to assess the heterogeneity across studies. Prediction intervals (PI) were computed to quantify the dispersion (or distribution) of effect estimates [42]. Funnel plot and Egger’s test [43] were used to evaluate publication bias. A *p*-value ≤ 0.05 was considered as significant difference. Publication bias across studies was assessed using funnel plots using standard error (SE) as an indicator of study size on the vertical axis and MD on the horizontal axis. Using subgroup analysis and meta-regression, participants’ characteristics (sex, age, and baseline BP and SC values), forest therapy procedure (session and duration), and design (RCT or Non-RCT) were analyzed for finding the cause of heterogeneity. All meta-analyses mentioned above were performed using the statistical analysis software (Stata/MP 17, StataCorp, College Station, TX, USA). To evaluate the stability of the overall estimate, sensitivity analyses were conducted with an open-source Python module of Meta-Analysis called “PythonMeta 1.26” [44]. The results are presented in Supplementary Tables S4–S6.

## 3. Results

### 3.1. Study Selection Process

**Figure 1:**
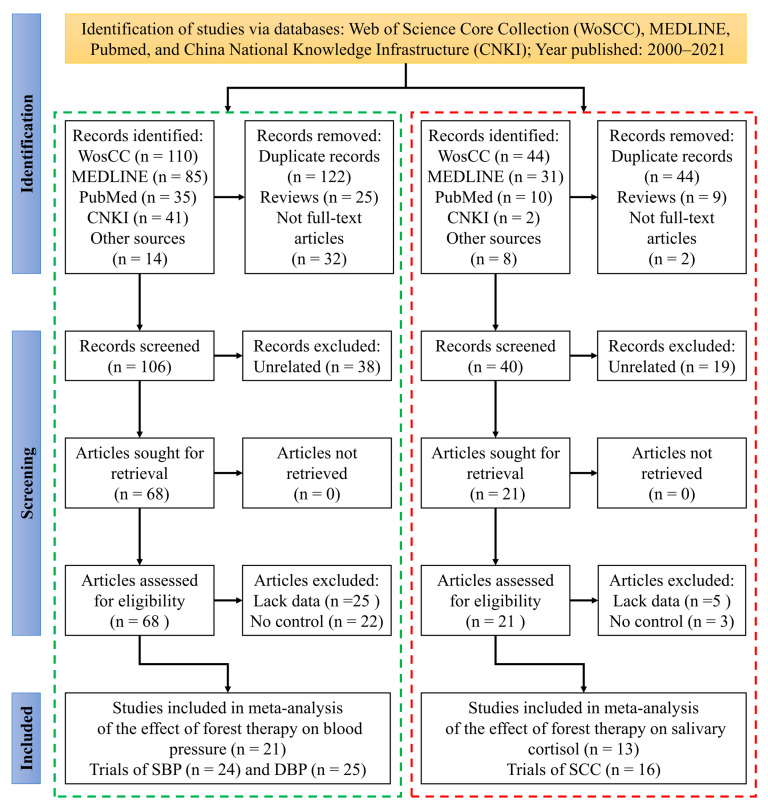
Flow diagram of the study selection process for the present study. This flow diagram was performed according to the Preferred Reporting Items for Systematic Reviews and Meta-Analyses (PRISMA) 2020 guidelines [33]. “Other sources” refers to articles included in earlier meta-analyses [23,31]. SBP: Systolic blood pressure; DBP: Diastolic blood pressure; SCC: Salivary cortisol concentration.

The article screening and study selection process for BP and SC were separately performed according to the Preferred Reporting Items for Systematic Reviews and Meta-Analyses (PRISMA) 2020 guidelines [33]. The review flow chart is detailed in Figure 1. After identification and screening, a total of 68 articles on blood pressure were reviewed as full texts to be assessed for eligibility. Ultimately, 21 studies fulfilled the inclusion criteria. The major included studies report only one trial, while four articles report two trials (e.g., walking or viewing). Additionally, one included study does not provide the SBP data, but only the DBP data. In total, 25 trials from 21 studies involving DBP were included in the meta-analysis, and 24 trials from 20 studies involving SBP. As for SCC, 13 studies met the inclusion criteria after identification, screening, and eligibility assessment. In these studies, 10 reported only one trial and 3 reported two trials. Finally, 16 trials were included in the meta-analysis of SCC.

### 3.2. Study Characteristics and Trial Information

Table 2 summarizes the key characteristics of the included studies. In the studies on SBP (*n* = 20), 4 are from Europe, and 16 originate in Asia; 2 were designed using Non-RCT, and 18 were designed using RCT; 14 recruited young or middle-aged participants (range 19–60 years), and 6 recruited older participants (age > 60 years) or mixed groups; the participants were all male in 5 studies and female in 4 studies, 8 studies had both male and female participants, and 3 studies did not provide participants’ sex information; 13 recruited young or middle-aged participants (ranging from 19–60 years), and 7 recruited older participants (age > 60 years) or mixed groups. Among 24 trials on SBP, 7 involved seated viewing, and 13 involved walking or multi-session programs; the duration of 11 trials was <20 min, and that of 13 trials was ≥20 min.

In terms of DBP studies (*n* = 21), 4 are from Europe, and 17 originate in Asia; 2 were designed using Non-RCT, and 19 were designed using RCT; 15 recruited young or middle-aged participants (ranging from 19–60 years), and 6 recruited older participants (age > 60 years) or mixed groups; the participants were all male in 6 studies and female in 4 studies, 8 studies had both male and female participants, and 3 studies did not provide participants’ sex information; 14 recruited young or middle-aged participants (ranging from 19–60 years), and 7 recruited older participants (age > 60 years) or mixed groups. In 25 trials on DBP, 8 involved seated viewing, and 17 involved walking or multi-session programs; the duration of 12 trials was <20 min, and that of 13 trials was ≥20 min.

The studies on SCC (*n* = 13) are mostly from Asia (*n* = 11), with 2 from Europe; most of the studies (*n* = 11) recruited young or middle-aged participants, except for 2 studies that recruited older participants; most participants in SCC studies (*n* = 10) were male, and 3 studies recruited mixed groups; as opposed to 3 Non-RCT trials, most (*n* = 10) studies used RCT designs. Among 16 trials on SCC, 8 studies involved seated viewing, and 8 involved walking or multi-session programs; the duration of 10 trials was <20 min, and that of 6 trials was ≥20 min.

### 3.3. Risk of Bias

In total, 10 parallel RCT studies were evaluated using the RoB 2 tool (see Figure S1); 15 crossover RCT studies were evaluated using the Excel tool to implement RoB 2 (see Figure S2); 4 non-RCT studies were evaluated using the ROBINS-I tool (see Figure S3). These tools judge the study as having a high risk of bias for at least one domain, or as having some concerns for multiple domains that significantly lower confidence in the results. All the parallel RCT studies were judged to have a high risk of bias because they were judged to have some concerns for multiple domains: the randomization process (*n* = 9), deviations from intended intervention (*n* = 7), missing outcome data (*n* = 2), measurements of the outcome (*n* = 10), and selection of the reported result (*n* = 10). As well, one of them was identified as a “serious” risk in the domain of the randomization process. For crossover RCT studies, some domains, including the randomization process (*n* = 7), deviations from intended intervention (*n* = 3), and missing outcome data (*n* = 2), were marked as a “serious” risk; multiple domains, including the randomization process (*n* = 8), period and carryover effects (*n* = 15), deviations from intended intervention (*n* = 10), missing outcome data (*n* = 1), measurements of the outcome (*n* = 15, and selection of the reported result (*n* = 15), were marked as being of “some concern.” Accordingly, all crossover RCT studies were considered to present a high risk of bias.

### 3.4. Meta-Analysis

**Figure 2:**
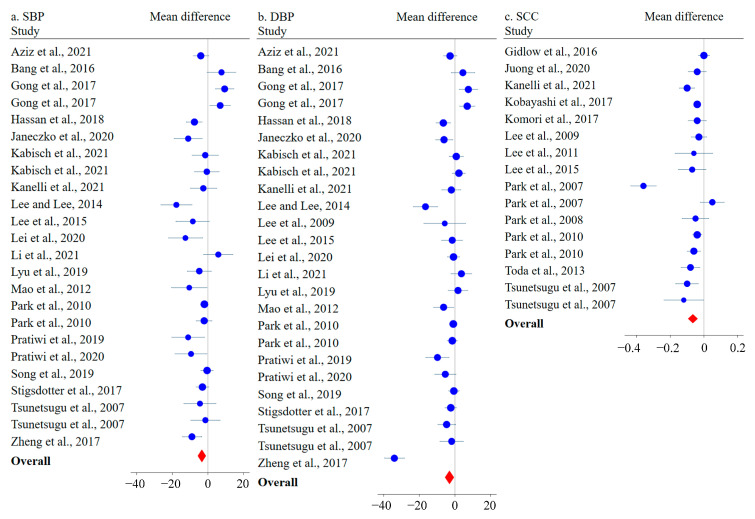
Forest plots referred to the changes in SBP (**a**), DBP (**b**), and SCC (**c**) between forest therapy and urban control, based on meta-analyses [45,46,47,48,49,50,51,52,53,54,55,56,57,58,59,60,61,62,63,64,65,66,67,68,69,70,71,72,73]. The blue dots indicate the effect values for individual studies and the red rhombuses indicate the total effect values.

**Figure 3:**
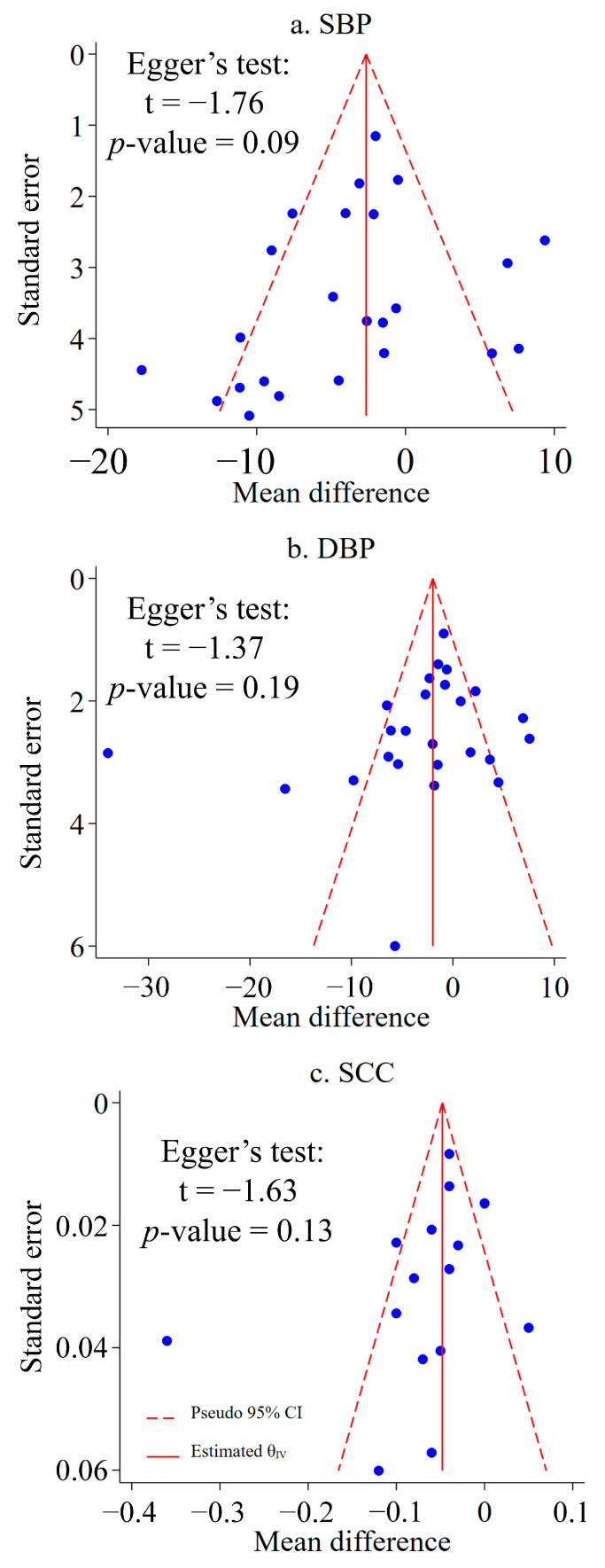
Funnel plots and Egger’s test for visual assessments of publication bias in the meta-analyses of forest therapy’s effects on SBP (**a**), DBP (**b**), and SCC (**c**) in urban residents. Blue symbols indicate included studies.

The meta-analysis estimates are outlined in Table 3 and Figure 2 (details can be found in the Supplementary Figures S4–S6). Statistically significant health-denoting associations between forest therapy and urban control groups were identified for SBP (*p* < 0.01), DBP (*p* = 0.02), and SCC (*p* < 0.01). The meta-analysis results showed that forest therapy was associated with decreased SBP −3.44 (95% CI −5.74, −1.14), DBP −3.07 (95% CI −5.59, −0.54), and SCC −0.07 (95% CI −0.10, −0.04). Among these meta-analyses, the τ^2^ scores for SBP (21.2229), DBP (34.7231), and SCC (0.0026), and the *I*^2^ scores for SBP (72.87%), DBP (88.59%), and SCC (83.85%), are all >60%, which indicates substantial heterogeneity. The 95% PI ranged from −13.30 to 6.42 for SBP, from −15.54 to 9.41 for DBP, and from −0.18 to 0.05 for SCC. The PIs for SBP, DBP, and SCC all contain values above 0. This means that, although forest therapy interventions were effective in all three outcomes, some of these interventions may not be effective. Furthermore, all three PIs contain largely negative values, suggesting that some interventions could have a substantial impact on SBP, DBP, and SCC. By analyzing funnel plots, most of the studies were found to be visually symmetric, with a narrow spread as the top (Figure 3). The results of Egger’s test (SBP: *p* = 0.09; DBP: *p* = 0.19; SCC: *p* = 0.13) on funnel asymmetry did not reach significant levels, demonstrating that publication bias had little effect on the overall results of SBP (Figure 3a), DBP (Figure 3b), and SCC (Figure 3c).

### 3.5. Subgroup Analysis

The studies were divided into three subgroups: the RCT group and non-RCT group (based on design), seated viewing group and walking or multi-session group (based on session), and <20 min group and ≥20 min group (based on duration). The subgroup analysis results of the design, session, and duration for SBP, DBP, and SCC are shown in Table 3 (details can be found in Supplementary Figures S7–S15). For SBP, DBP, and SCC, the differences between the groups divided by design (*p* = 0.63–0.79), session (*p* = 0.16–0.36), and duration (*p* = 0.16–0.79) are not significant. Despite excluding the non-RCT group from the subgroup analyses of SBP, DBP, and SCC, substantial heterogeneity (*I*^2^ = 75.21–89.53%) was still found in the RCT group. The seated viewing group (*I*^2^ = 60.96–89.80%) and the walking or multi-session group (*I*^2^ = 70.17–91.15%) both have the *I*^2^ scores of >60% in the subgroup analyses of SBP, DBP, and SCC. When studies with the duration of <20 min were excluded, substantial heterogeneity (*I*^2^ = 82.49–94.18%) was still observed in the ≥20 min group. It is suggested that study design, session, and duration do not contribute to heterogeneity in the effects of forest therapy on SBP, DBP, and SCC outcomes.

Further, subgroup analyses indicated that both the seated viewing group and walking or multi-session group exhibited significant impacts (*p* < 0.05) on SBP, DBP, and SCC, suggesting that forest therapy sessions have no obvious differences in their health effects on BP and SC. We also found that forest therapy programs lasting for ≥20 min exhibited significant effects (*p* < 0.01) on SBP, DBP, and SCC, whereas those lasting for <20 min only had significant effects on DBP (*p* = 0.02). This finding demonstrates that forest therapy programs lasting for ≥20 min have greater health effects on BP and SC, compared with those lasting for <20 min.

### 3.6. Meta-Regression Analysis

**Figure 4:**
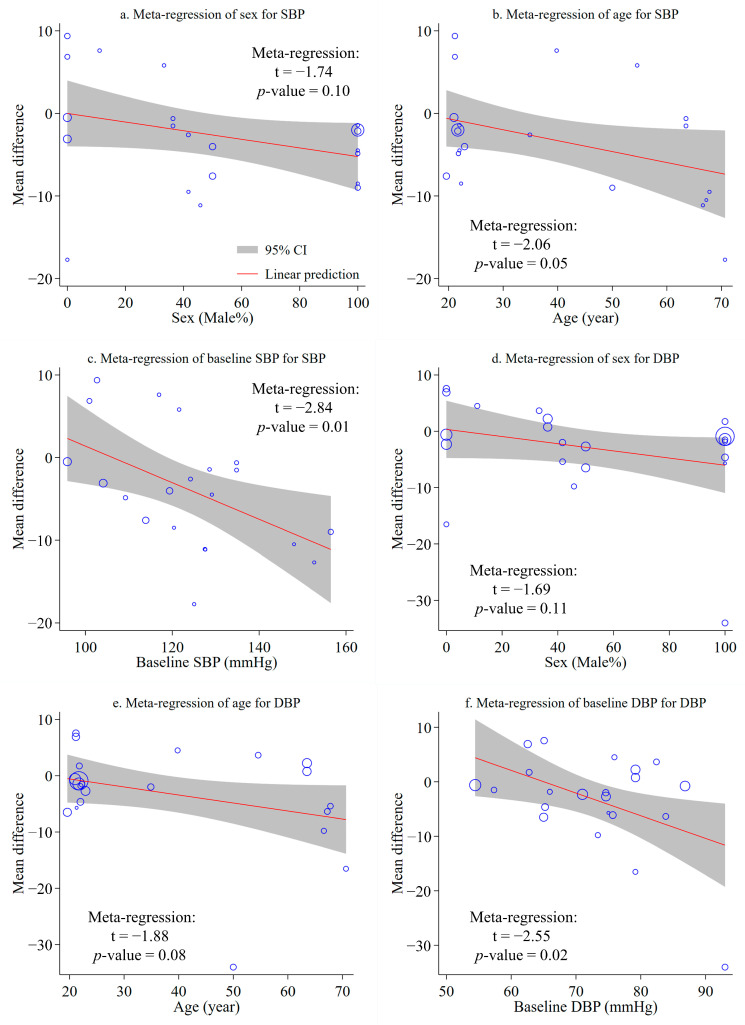
Bubble plots and meta-regressions for visual assessments of the effects of sex, age, and baseline blood pressure on the mean difference between SBP and DBP. Blue symbols indicate included studies.

Table S3 shows a meta-regression analysis of participants’ sex, age, and baseline values (SBP, DBP, and SCC) for the MD values of SBP, DBP, and SCC. The results indicated that participants’ sex (*p* = 0.72), age (*p* = 0.89), and baseline SCC (*p* = 0.74) were not significantly correlated to SCC outcomes. Additionally, participants’ sex, age, and baseline SBP and DBP had different impacts on the MD values of SBP and DBP. As shown in Figure 4, the health effects (reducing BP and SC) of forest therapy generally increased as participants’ sex, age, and baseline SBP and DBP increased. It was observed that only the correlations between SBP outcomes and participants’ age (*p* = 0.05; Figure 4b) and baseline SBP (*p* = 0.01; Figure 4c), as well as between the DBP MD values and baseline DBP (*p* = 0.02; Figure 4f), were statistically significant. Yet, participants’ sex is not significantly associated with SBP (*p* = 0.10, Figure 4a) or DBP (*p* = 0.11, Figure 4d), and participants’ age is not significantly associated with the DBP MD values (*p* = 0.08, Figure 4e). Accordingly, these findings suggest that participants’ age and baseline SBP are the primary contributors to heterogeneity in forest therapy’s impact on SBP outcomes; participants’ baseline DBP is the most important contributor to heterogeneity in forest therapy’s impact on DBP outcomes. However, the sources of heterogeneity in forest therapy’s impacts on SCC outcomes have not been identified.

## 4. Discussion

### 4.1. Health Benefits of Forest Therapy to BP and Mental Stress

Spending time outside, especially in environments with green space, has been shown to reduce stress and ultimately improve health [74,75]. In previous meta-analyses, Yao et al. [35] reported that nature exposure significantly reduced participants’ SBP, DBP, and SCC outcomes, with MD values of −3.82 [95% CI −6.77, −0.86], −3.17 [95% CI −6.01, −0.33], and −0.06 [95% CI −0.08, −0.04], respectively; Ideno et al. [31] and Antonelli et al. (2019) also found that forest bathing significantly decreased SBP (−3.15 [95% CI −4.12, −2.18]), DBP (−1.75 [95% CI −2.38, −1.13]), and SCC (−0.05 [95% CI −0.06, −0.04]). Similarly, our meta-analyses reveal that forest therapy significantly lowers participants’ SBP, DBP, and SCC when compared to urban control. Following these results, derived from sufficient trials, we can obtain a reliable answer to the first question regarding forest therapy’s capability to lower blood pressure and reduce stress in urban populations. The forest therapy remarkably improved cardiovascular function, hemodynamic indexes, neuroendocrine indexes, and metabolic indexes [17,76], as well as having positive impacts on anxiety, depression, anger, fatigue, and confusion [77,78,79]. According to these findings, forest therapy is believed to have an antistress component associated with its “anticipatory effect” on cortisol levels [80], and to have a beneficial effect on BP through the phytoncides’ function [81], autonomic nervous system’ regulation [31], and other mechanisms. Further, these findings provide theoretical support for the connection between forest therapy and human physiology and emphasize forest therapy’s importance in controlling blood pressure and managing stress.

### 4.2. Heterogeneity and Its Cause

Statistical tests for heterogeneity provided an answer to the second question: substantial heterogeneity was observed in the meta-analyses of forest therapy’s effects on SBP, DBP, and SCC outcomes. Similar results were also observed in Yao’s study [35] regarding the SBP (*I*^2^ = 88%) and DBP (*I*^2^ = 88%), as well as Antonelli’s study [23] on SCC (*I*^2^ = 88%), whereas the SCC (*I*^2^ = 22%) meta-analysis in Yao’s study [35] or the meta-analyses of SBP (*I*^2^ = 1%) and DBP (*I*^2^ = 24%) in Ideno’s study [31] had no heterogeneity. The variations can be attributed to the different search strategies (database and search query), selection of studies (identification and screening), intervention type (forest therapy vs. forest bathing vs. nature exposure), year published (2017–2022), number of studies included (7–25), and number of participants (705–2270). On the other hand, for SBP, DBP, and SCC, the PIs were substantially wider than the CIs and contained values above 0, thus providing high confidence in our findings that forest therapy interventions decrease SBP, DBP, and SCC on average, as well as motivating researchers to conduct further studies to determine which type of intervention is the most effective.

The subgroup analysis revealed that neither the study design nor the intervention procedure (session and duration) contributed to the heterogeneity in the meta-analyses of SBP, DBP, and SCC. It appears that study design and intervention procedure are not the primary factors responsible for forest therapy’s effects on BP and SC. If we continued to analyze the subgroups, we found that interventions lasting for ≥20 min had greater effects on BP and SC than those lasting for <20 min. Possibly, this is due to participants’ deep connection with forests through their five senses [23], which may take more time to manifest. Hence, we suggested that the forest therapy procedure should be designed with a longer duration of ≥20 min. A similar finding was also observed in Hunter’s study [82] on the nature experience’s effects on the levels of SC and alpha-amylase, which suggests that, when the duration of the nature experience varies between 20 and 30 min, the benefits accrue more effectively. Also, similarly to walking or multi-session programs, seated viewing had a significant health effect on BP (reduction of SBP and DBP) and mental stress (reduction of SCC). In agreement with Hunter’s study [82], the type of nature-based activity did not affect the cortisol response. Likewise, Ideno et al. [31] reported that the impact of the forest environment on BP was not directly related to physical activity (i.e., walking group versus non-walking group).

Forest environments have a greater effect on lowering SBP in older people than in younger people and in hypertensive people than in normotensive people [31]. Using meta-regression analyses, we also found that the SBP outcomes of participants in the forest therapy group decreased more as participants’ age and baseline SBP increased. Their significant correlation manifested that participants’ age and baseline SBP were probably the primary sources of heterogeneity in the meta-analyses of SBP. According to the general consensus, older age increased the probability of hypertension [15,83], which explains why forest therapy reduced blood pressure considerably in the elderly. We also found that DBP reduction in the forest therapy group was positively correlated with their baseline values, supporting that the baseline DBP is a primary influence on forest therapy’s effects on DBP outcomes. Further, we observed that participant groups with a higher percentage of males performed better in terms of SBP and DBP reduction, as well as that those with older ages performed better in terms of DBP reduction after forest therapy. The non-significant correlations among the variables indicate that the percentage of males may be a contributor to heterogeneity in the meta-analysis of SBP, and both the percentage of males and age may contribute to heterogeneity in the meta-analysis of DBP. These observations have partially answered the third question, suggesting that the elderly and/or the hypertensive ought to participate in forest therapy programs to lower their blood pressure. Notably, we did not detect any significant contributors to the heterogeneity in the meta-analysis of forest therapy’s impact on SCC outcomes, thus no reliable information could be obtained to answer the third question. There is a possibility that there are few studies on this topic, resulting in insufficient subgroup analyses and meta-regressions.

These findings provide a theoretical explanation for the potential causes of heterogeneity among studies of forest therapy’s effects on BP, and provide effective guidance to design forest therapy programs, such as controlling duration sessions. Moreover, our findings are useful in identifying the participants who are most likely to benefit from forest therapy.

### 4.3. Limitations

Although sufficient analyses were performed, there are some limitations. First of all, only the literature from WoSCC, MEDLINE, PubMed, and CNKI were considered, which resulted in some essential information from other databases being left out. In addition, the major included studies are designed by RCT, yet these environmental interventions cannot be blind to the therapist and the participants. Consequently, all studies pose a high risk of bias due to randomization, deviation from intended intervention, or measurement of the outcome. Despite no significant publication biases being observed, we recommend caution with regard to our conclusions. Furthermore, in some included studies, certain critical data (such as participants’ age or sex) are not described, which meant subgroup and meta-regression analyses could not be performed in accordance with conventional procedures. Also, a majority of the included studies fail to provide the key data on changes in outcomes between the final value and the baseline value. As a result, we are only able to assess the effects of forest therapy using the final outcome value. Although the results of this meta-analysis remain valid and reliable, the accuracy should be regarded cautiously.

### 4.4. Future Research

In view of the fact that there is limited research on SCC and that the findings are limited in analyzing its relationship with forest therapy, future research on the effects of forest therapy on human health should pay attention to SCC outcomes. Additionally, in future trials of the effects of forest therapy on BP outcomes, participants’ characteristics, such as their age and baseline BP, should be primarily considered. Furthermore, we only surveyed the intervention procedure and participants’ characteristics, rather than the forest characteristics, such as forest types or seasonality, which have proven to be related to greater variability in the characteristics of negative air ion concentration, air oxygen content, human comfort index, and phytoncide relative content [84]. Hence, these forest characteristics should be investigated in future research to understand why forest therapy has such heterogeneous effects on human health. As reported by Kim and Shin [85], guided forest therapy differs from self-guided forest therapy in the healing factors and health benefits, suggesting that future research should also consider the possibility of performing forest therapy alone or with a guide when identifying the cause of heterogeneity among studies. Another suggestion is that future studies should be undertaken internationally in order to assess whether forest therapy is beneficial to a greater number of individuals from various regions.

## 5. Conclusions

In summary, meta-analyses suggest that forest therapy reduces BP and relieves stress (reducing SCC) in urban residents, although there is a high degree of heterogeneity throughout. Various attributes of participants’ sex, age, and baseline BP levels determine the heterogeneity in the BP-lowering effects of forest therapy. Furthermore, those participants who are older or who have higher baseline BP levels exhibit greater BP-lowering effects after forest therapy intervention. We also found that forest therapy programs lasting for longer durations (≥20 min) have greater BP- and SCC-lowering effects on participants, compared with those lasting for shorter duration (<20 min). Forest therapy sessions such as viewing, walking, or multi-session programs have similar health effects on BP reduction and stress relief. Our findings further support forest environments’ therapeutic effects on human health, recommending that urban residents spend enough time outdoors in natural settings such as forests. However, this study failed to identify the causes of heterogeneity among forest therapy programs for lowering levels of SCC, and further research is required for targeted treatment.

## Figures and Tables

**Table 1:** Inclusive criteria for the meta-analysis.

Criteria	Inclusion
P (Participants)	Adults living in urban areas, regardless of their health status
I (Intervention)	All types of forest therapy activities (real forest-based seated viewing, walking, or multi-session program)
C (Comparison)	Visiting urban environments (urban environment-based seated viewing, walking, or multi-session program)
O (Outcomes)	Measurement of the participants’ SBP, and/or DBP, and/or SCC after intervention

**Table 2:** Key characteristics of 29 included studies.

Reference	Design (RCT/Non-RCT)	Study Location	Participants’ Characteristics		Intervention Procedure (Sessions and Duration)	Outcomes		
			Sex; Male%	Mean Age (SD) or Range; Year		SBP	DBP	SCC
Abdul Aziz et al., 2021 [45]	Crossover RCT	Malaysia	50	22.93 (1.0); 20–25	Forest walking (20 min)	○	○	
Bang et al., 2016 [46]	Parallel RCT	Korea	7.4	39.8	Forest walking (5 weeks)	○	○	
Gidlow et al., 2016 [47]	Crossover RCT	United Kingdom	65	47.9 (11.6)	Forest walking (30 min)			○
Gong et al., 2017 [48]	Parallel RCT	China	0	21.17 (1.46); 19–24	1. Seated viewing (30 min)	○	○	
					2. Forest walking (60 min)			
Hassan et al., 2018 [49]	Crossover RCT	China	50	19.6 (1.42); 19–24	Forest walking (15 min)	○	○	
Janeczko et al., 2020 [50]	Parallel RCT	Poland	/	19–24	Forest walking (30 min)	○	○	
Joung et al., 2020 [51]	Non-RCT	Korea	62.5	20.9 (1.3)	Forest walking (15 min)			○
Kabisch et al., 2021 [52]	Parallel RCT	Germany	36.4	63.5 (4.2); 55–70	1. Seated viewing (15 min)	○	○	
					2. Seated viewing (15 min) + walking (30 min)			
Kanelli et al., 2021 [53]	Non-RCT	Greece	41.7	34.9 (11.0)	Forest walking (60 min)	○	○	○
Kobayashi et al., 2017 [54]	Crossover RCT	Japan	0	70.7; 60–80	Seated viewing (15 min)			○
Komori et al., 2017 [55]	Crossover RCT	Japan	100	31.5 (5.6)	Forest walking (2 h)			○
Lee and Lee, 2014 [56]	Parallel RCT	Japan	0	70.65; 60–80	Forest walking (60 min)	○	○	
Lee et al., 2009 [57]	Crossover RCT	Japan	100	21.3 (1.1); 20–23	Seated viewing (15 min)		○	○
Lee et al., 2011 [58]	Cross-over RCT	Japan	100	21.2 (0.9)	Seated viewing (15 min)			○
Lee et al., 2015 [59]	Crossover RCT	Japan	100	22.3 (1.3)	Seated viewing (15 min)	○	○	○
Lei et al., 2020 [60]	Parallel RCT	China	/	60–70	Forest bathing program (5 days)	○	○	
Li et al., 2020 [61]	Parallel RCT	China	33.3	54.56; 40–71	Forest walking (15 min)	○	○	
Lyu et al., 2019 [62]	Parallel RCT	China	100	21.7; 19–24	Forest therapy program (3 days)	○	○	
Mao et al., 2012 [63]	Parallel RCT	China	/	66.6; 60–75	Forest bathing program (7 days)	○	○	
Park et al., 2007 [64]	Crossover RCT	Japan	100	22.8 (1.4)	1. Seated viewing (20 min)			○
					2. Forest walking (20 min)			
Park et al., 2008 [65]	Crossover RCT	Japan	100	21.3 (1.1)	Seated viewing (15 min)			○
Park et al., 2010 [66]	Crossover RCT	Japan	100	21.7 (1.5)	1. Seated viewing (average 14 min)	○	○	○
					2. Forest walking (average 16 min)			
Pratiwi et al., 2019 [67]	Crossover RCT	Japan	45.8	66.6	Seated viewing (11–15 min)	○	○	
Pratiwi et al., 2020 [68]	Crossover RCT	Japan	41.7	67.8	Forest walking (11–15 min)	○	○	
Song et al., 2019 [69]	Crossover RCT	China	0	21.0 (1.3)	Seated viewing (15 min)	○	○	
Stigsdotter et al., 2017 [70]	Non-RCT	Denmark	0	20–36	Seated viewing (50 min) + walking (15 min)	○	○	
Toda et al., 2013 [71]	Non-RCT	Japan	100	67.6 (2.8); 64–74	Seated viewing (45 min)			○
Tsunetsugu et al., 2007 [72]	Crossover RCT	Japan	100	22.0 (1.0); 21–23	1. Seated viewing (15 min)	○	○	○
					2. Forest walking (15 min)			
Zheng et al., 2017 [73]	Parallel RCT	China	100	50	Forest bathing program (20 days)	○	○	

The “○” symbol indicates that an outcome was measured in the included study.

**Table 3:** Summary meta-analysis result of the mean difference (MD) between forest therapy and urban control groups. CI: Confidence interval; PI: Prediction interval; *I*^2^: I-squared statistics; τ^2^: Tau-squared statistics.

Outcomes	Subgroup Analysis	Number of Studies	Number of Participants	Effect MD (95% CI)	95% PI	Heterogeneity (τ^2^)	Heterogeneity (*I*^2^; %)	*p*-Value
SBP	Overall	24	2246	−3.44 (−5.74, −1.14)	(−13.30, 6.42)	21.2229	72.87	<0.01
	Design-based subgroup							0.79
	RCT	22	2096	−3.55 (−6.12, −0.99)			75.21	<0.01
	Non-RCT	2	150	−3.00 (−6.21, −0.20)			0.00	0.90
	Session-based subgroup							0.36
	Seated viewing	7	896	−1.89 (−5.23, 1.46)			60.96	0.02
	Walking or multi-session program	17	1350	−4.04 (−7.14, −0.94)			75.80	<0.01
	Duration-based subgroup							0.53
	<20 min	11	1292	−2.72 (−4.84, −0.60)			37.87	0.10
	≥20 min	13	954	−4.25 (−8.47, −0.02)			82.49	<0.01
DBP	Overall	25	2270	−3.07 (−5.59, −0.54)	(−15.54, 9.41)	34.7231	88.59	0.02
	Design-based subgroup							0.63
	RCT	23	2120	−3.17 (−5.94, −0.39)			89.53	<0.01
	Non-RCT	2	150	−2.22 (−4.96, 0.52)			0.00	0.92
	Session-based subgroup							0.25
	Seated viewing	8	920	−1.13 (−3.80, 1.54)			69.08	<0.01
	Walking or multi-session program	17	1350	−3.81 (−7.51, −0.11)			91.15	<0.01
	Duration-based subgroup							0.35
	<20 min	12	1316	−1.60 (−3.33, 0.12)			52.77	0.02
	≥20 min	13	954	−4.18 (−9.33, 0.98)			93.42	<0.01
SCC	Overall	16	1786	−0.07 (−0.10, −0.04)	(−0.18, 0.05)	0.0026	83.85	<0.01
	Design-based subgroup							0.67
	RCT	13	1654	−0.06 (−0.10, −0.03)			86.02	<0.01
	Non-RCT	3	132	−0.08 (−0.11, −0.04)			30.66	0.24
	Session-based subgroup							0.16
	Seated viewing	8	1346	−0.09 (−0.14, −0.04)			89.80	<0.01
	Walking or multi-session program	8	440	−0.05 (−0.08, −0.01)			70.17	<0.01
	Duration-based subgroup							0.36
	<20 min	10	1538	−0.04 (−0.06, −0.03)			0.00	0.74
	≥20 min	6	248	−0.09 (−0.17, 0.00)			94.18	<0.01

## Data Availability

All datasets presented in this study can be found within the article and in the Supplementary Materials.

## Supplementary Materials

The following supporting information can be downloaded at: https://www.mdpi.com/article/10.3390/ijerph20010458/s1, Figure S1: Risk of bias graph for parallel randomized controlled trials studies; Figure S2: Risk of bias graph for crossover randomized controlled trials studies; Figure S3: Risk of bias graph for non-randomized controlled trials studies; Figure S4: Meta-analysis of the effects of forest therapy and urban control on systolic blood pressure; Figure S5: Meta-analysis of the effects of forest therapy and urban control on diastolic blood pressure; Figure S6: Meta-analysis of the effects of forest therapy and urban control on salivary cortisol concentration; Figure S7: Study design-based subgroup analysis of the effects of forest therapy and urban control on systolic blood pressure; Figure S8: Session-based subgroup analysis of the effects of forest therapy and urban control on systolic blood pressure; Figure S9: Duration-based subgroup analysis of the effects of forest therapy and urban control on systolic blood pressure; Figure S10: Study design-based subgroup analysis of the effects of forest therapy and urban control on diastolic blood pressure; Figure S11: Session-based subgroup analysis of the effects of forest therapy and urban control on diastolic blood pressure; Figure S12: Duration-based subgroup analysis of the effects of forest therapy and urban control on diastolic blood pressure; Figure S13: Study design-based subgroup analysis of the effects of forest therapy and urban control on salivary cortisol concentration; Figure S14: Session-based subgroup analysis of the effects of forest therapy and urban control on salivary cortisol concentration; Figure S15: Duration-based subgroup analysis of the effects of forest therapy and urban control on salivary cortisol concentration; Table S1: PRISMA checklist; Table S2: Search strategies for all databases searched; Table S3: Results of meta-regression; Table S4. Sensitivity analysis of the effects of forest therapy and urban control on systolic blood pressure; Table S5. Sensitivity analysis of the effects of forest therapy and urban control on diastolic blood pressure; Table S6. Sensitivity analysis of the effects of forest therapy and urban control on salivary cortisol concentration.
