# Supplementary material for: The Effects of Forest Therapy on the Blood Pressure and Salivary Cortisol Levels of Urban Residents: A Meta-Analysis
Source: Int J Environ Res Public Health. 2022 Dec 27;20(1):458. doi: 10.3390/ijerph20010458 (PMC9819785; doi:10.3390/ijerph20010458)
Supplement: Supplementary file 1 [file ijerph-20-00458-s001.zip › ijerph-1974100-supplementary.pdf]

# SUPPLEMENTARY MATERIALS

## Contents of tables


Table S1. PRISMA checklist ……………………………………………………………………………………………...1

Table S2. Search strategies for all databases searched…………………………………………………………………………….3

Table S3. Results of meta-regression………………………………………………………………………………………………..3

Table S4. Sensitivity analysis of the effects of forest therapy and urban control on systolic blood pressure……………………………..4

Table S5. Sensitivity analysis of the effects of forest therapy and urban control on diastolic blood pressure…………………………...5

Table S6. Sensitivity analysis of the effects of forest therapy and urban control on salivary cortisol concentration……...………………..6


## Contents of figures


Figure S1. Risk of bias graph for parallel randomized controlled trials studies ......................................................7

Figure S2. Risk of bias graph for crossover randomized controlled trials studies ......................................................8

Figure S3. Risk of bias graph for non-randomized controlled trials studies……………………………………………...…………....9

Figure S4. Meta-analysis of the effects of forest therapy and urban control on systolic blood pressure......................................................10

Figure S5. Meta-analysis of the effects of forest therapy and urban control on diastolic blood pressure ....................................................11

Figure S6. Meta-analysis of the effects of forest therapy and urban control on salivary cortisol concentration .........................................12

Figure S7. Study design-based subgroup analysis of the effects of forest therapy and urban control on systolic blood pressure………....13

Figure S8. Session-based subgroup analysis of the effects of forest therapy and urban control on systolic blood pressure………………..14

Figure S9. Duration-based subgroup analysis of the effects of forest therapy and urban control on systolic blood pressure……………...15

Figure S10. Study design-based subgroup analysis of the effects of forest therapy and urban control on diastolic blood pressure…...….16

Figure S11. Session-based subgroup analysis of the effects of forest therapy and urban control on diastolic blood pressure……..……...17

Figure S12. Duration-based subgroup analysis of the effects of forest therapy and urban control on diastolic blood pressure……...….18

Figure S13. Study design-based subgroup analysis of the effects of forest therapy and urban control on salivary cortisol concentration.19

Figure S14. Session-based subgroup analysis of the effects of forest therapy and urban control on salivary cortisol concentration…...…20

Figure S15. Duration-based subgroup analysis of the effects of forest therapy and urban control on salivary cortisol concentration……..21

## Table S1. PRISMA checklist

| Section and Topic | Item # | Checklist item | Location where item is reported |
|---|---|---|---|
| **TITLE** | | | |
| Title | 1 | Identify the report as a systematic review. | Page 1 |
| **ABSTRACT** | | | |
| Abstract | 2 | See the PRISMA 2020 for Abstracts checklist. | Page 1 |
| **INTRODUCTION** | | | |
| Rationale | 3 | Describe the rationale for the review in the context of existing knowledge. | Page 2 |
| Objectives | 4 | Provide an explicit statement of the objective(s) or question(s) the review addresses. | Page 2 |
| **METHODS** | | | |
| Eligibility criteria | 5 | Specify the inclusion and exclusion criteria for the review and how studies were grouped for the syntheses. | Page 3 |
| Information sources | 6 | Specify all databases, registers, websites, organisations, reference lists and other sources searched or consulted to identify studies. Specify the date when each source was last searched or consulted. | Page 3 |
| Search strategy | 7 | Present the full search strategies for all databases, registers and websites, including any filters and limits used. | Page 3 |
| Selection process | 8 | Specify the methods used to decide whether a study met the inclusion criteria of the review, including how many reviewers screened each record and each report retrieved, whether they worked independently, and if applicable, details of automation tools used in the process. | Page 3 |
| Data collection process | 9 | Specify the methods used to collect data from reports, including how many reviewers collected data from each report, whether they worked independently, any processes for obtaining or confirming data from study investigators, and if applicable, details of automation tools used in the process. | Page 4 |
| Data items | 10a | List and define all outcomes for which data were sought. Specify whether all results that were compatible with each outcome domain in each study were sought (e.g. for all measures, time points, analyses), and if not, the methods used to decide which results to collect. | Page 4 |
| | 10b | List and define all other variables for which data were sought (e.g. participant and intervention characteristics, funding sources). Describe any assumptions made about any missing or unclear information. | Page 4 |
| Study risk of bias assessment | 11 | Specify the methods used to assess risk of bias in the included studies, including details of the tool(s) used, how many reviewers assessed each study and whether they worked independently, and if applicable, details of automation tools used in the process. | Page 4 |
| Effect measures | 12 | Specify for each outcome the effect measure(s) (e.g. risk ratio, mean difference) used in the synthesis or presentation of results. | Pages 4–5 |
| Synthesis methods | 13a | Describe the processes used to decide which studies were eligible for each synthesis (e.g. tabulating the study intervention characteristics and comparing against the planned groups for each synthesis (item #5)). | Pages 4–5 |
| | 13b | Describe any methods required to prepare the data for presentation or synthesis, such as handling of missing summary statistics, or data conversions. | Pages 4–5 |
| | 13c | Describe any methods used to tabulate or visually display results of individual studies and syntheses. | Pages 4–5 |
| | 13d | Describe any methods used to synthesize results and provide a rationale for the choice(s). If meta-analysis was performed, describe the model(s), method(s) to identify the presence and extent of statistical heterogeneity, and software package(s) used. | Pages 4–5 |
| | 13e | Describe any methods used to explore possible causes of heterogeneity among study results (e.g. subgroup analysis, meta-regression). | Pages 4–5 |
| | 13f | Describe any sensitivity analyses conducted to assess robustness of the synthesized results. | Pages 4–5 |
| Reporting bias assessment | 14 | Describe any methods used to assess risk of bias due to missing results in a synthesis (arising from reporting biases). | Page 5 |
| Certainty assessment | 15 | Describe any methods used to assess certainty (or confidence) in the body of evidence for an outcome. | Page 5 |

| Section and Topic | Item # | Checklist item | Location where item is reported |
|---|---|---|---|
| **RESULTS** | | | |
| Study selection | 16a | Describe the results of the search and selection process, from the number of records identified in the search to the number of studies included in the review, ideally using a flow diagram. | Pages 5–6 |
| | 16b | Cite studies that might appear to meet the inclusion criteria, but which were excluded, and explain why they were excluded. | Pages 5–6 |
| Study characteristics | 17 | Cite each included study and present its characteristics. | Pages 5, 7–8 |
| Risk of bias in studies | 18 | Present assessments of risk of bias for each included study. | Page 9 |
| Results of individual studies | 19 | For all outcomes, present, for each study: (a) summary statistics for each group (where appropriate) and (b) an effect estimate and its precision (e.g. confidence/credible interval), ideally using structured tables or plots. | Pages 9–11 |
| Results of syntheses | 20a | For each synthesis, briefly summarise the characteristics and risk of bias among contributing studies. | Pages 9–11 |
| | 20b | Present results of all statistical syntheses conducted. If meta-analysis was done, present for each the summary estimate and its precision (e.g. confidence/credible interval) and measures of statistical heterogeneity. If comparing groups, describe the direction of the effect. | Pages 9–11 |
| | 20c | Present results of all investigations of possible causes of heterogeneity among study results. | Pages 9–11 |
| | 20d | Present results of all sensitivity analyses conducted to assess the robustness of the synthesized results. | Supp |
| Reporting biases | 21 | Present assessments of risk of bias due to missing results (arising from reporting biases) for each synthesis assessed. | Page 12 |
| Certainty of evidence | 22 | Present assessments of certainty (or confidence) in the body of evidence for each outcome assessed. | Page 9 |
| **DISCUSSION** | | | |
| Discussion | 23a | Provide a general interpretation of the results in the context of other evidence. | Pages 14–15 |
| | 23b | Discuss any limitations of the evidence included in the review. | Page 15 |
| | 23c | Discuss any limitations of the review processes used. | Page 15 |
| | 23d | Discuss implications of the results for practice, policy, and future research. | Page 15 |
| **OTHER INFORMATION** | | | |
| Registration and protocol | 24a | Provide registration information for the review, including register name and registration number, or state that the review was not registered. | Not registered |
| | 24b | Indicate where the review protocol can be accessed, or state that a protocol was not prepared. | Not registered |
| | 24c | Describe and explain any amendments to information provided at registration or in the protocol. | Not registered |
| Support | 25 | Describe sources of financial or non-financial support for the review, and the role of the funders or sponsors in the review. | Page 16 |
| Competing interests | 26 | Declare any competing interests of review authors. | Page 16 |
| Availability of data, code and other materials | 27 | Report which of the following are publicly available and where they can be found: template data collection forms; data extracted from included studies; data used for all analyses; analytic code; any other materials used in the review. | Page 16 |

From: Page MJ, McKenzie JE, Bossuyt PM, Boutron I, Hoffmann TC, Mulrow CD, et al. The PRISMA 2020 statement: an updated guideline for reporting systematic reviews. BMJ 2021;372:n71. doi: 10.1136/bmj.n71

## Table S2. Search strategies for all databases searched

| Topic | Database | Search query |
|---|---|---|
| Blood pressure | WoSCC | TS = (forest* AND (urban* OR city*) AND “blood pressure”) |
| | MEDLINE | TS = (forest* AND (urban* OR city*) AND “blood pressure”) |
| | PubMed | ((forest*[Title/Abstract]) AND (urban*[Title/Abstract] OR city*[Title/Abstract])) AND ("blood pressure"[Title/Abstract]) |
| | CNKI | SU='forest'*'blood pressure' |
| Salivary cortisol | WoSCC | TS = (forest* AND (urban* OR city*) AND salivary cortisol*) |
| | MEDLINE | TS = (forest* AND (urban* OR city*) AND salivary cortisol*) |
| | PubMed | ((forest*[Title/Abstract]) AND (urban*[Title/Abstract] OR city*[Title/Abstract])) AND (salivary cortisol* [Title/Abstract]) |
| | CNKI | SU = 'forest'*'salivary cortisol' |

Databases include the Web of Science Core Collection (WoSCC), MEDLINE, PubMed, and China National Knowledge Infrastructure (CNKI).

## Table S3. Results of meta-regression

| Dependent variable | Independent variable | Number of observations | Coefficient | SE | t | *p*-value | 95%CI | |
|---|---|---|---|---|---|---|---|---|
| SBP | Sex | 21 | −0.05 | 0.03 | −1.74 | 0.10 | −0.12 | 0.01 |
| | Age | 21 | −0.13 | 0.06 | −2.06 | 0.05 | −0.27 | 0.00 |
| | Baseline SBP | 21 | −0.22 | 0.08 | −2.84 | 0.01 | −0.38 | -0.06 |
| DBP | Sex | 22 | −0.06 | 0.04 | −1.69 | 0.11 | −0.14 | 0.01 |
| | Age | 22 | −0.14 | 0.08 | −1.88 | 0.08 | −0.30 | 0.02 |
| | Baseline DBP | 22 | −0.42 | 0.16 | −2.55 | 0.02 | −0.76 | −0.08 |
| SCC | Sex | 16 | −0.00 | 0.00 | −0.36 | 0.72 | −0.00 | 0.00 |
| | Age | 16 | 0.00 | 0.00 | 0.15 | 0.89 | −0.00 | 0.00 |
| | Baseline SCC | 12 | −0.08 | 0.23 | −0.35 | 0.74 | −0.60 | 0.44 |

Results of meta-regression of participants’ sex, age, and baseline SBP, DBP, and SCC for SBP, DBP, and SCC outcomes are shown.

**Table S4. Sensitivity analysis of the effects of forest therapy and urban control on systolic blood pressure**

| Omitted study | Effect MD (95% CI) | Heterogeneity ($I^2$; %) |
|---|---|---|
| Aziz et al., 2021 | −3.50 [−5.94, −1.07] | 74.15 |
| Bang et al., 2016 | −3.87 [−6.18, −1.57] | 72.55 |
| Gong et al., 2017 | −4.09 [−6.19, −1.98] | 65.23 |
| Gong et al., 2017 | −3.98 [−6.23, −1.72] | 70.43 |
| Hassan et al., 2018 | −3.29 [−5.66, −0.91] | 72.64 |
| Janeczko et al., 2020 | −3.20 [−5.52, −0.88] | 72.72 |
| Kabisch et al., 2021 | −3.60 [−5.99, −1.22] | 74.23 |
| Kabisch et al., 2021 | −3.64 [−6.03, −1.26] | 74.15 |
| Kanelli et al., 2021 | −3.56 [−5.94, −1.17] | 74.26 |
| Lee and Lee, 2014 | −2.95 [−5.15, −0.74] | 69.64 |
| Lee et al., 2015 | −3.35 [−5.70, −1.00] | 73.81 |
| Lei et al., 2020 | −3.21 [−5.52, −0.90] | 72.92 |
| Li et al., 2021 | −3.84 [−6.16, −1.52] | 72.93 |
| Lyu et al., 2019 | −3.46 [−5.85, −1.07] | 74.13 |
| Mao et al., 2012 | −3.30 [−5.63, −0.96] | 73.52 |
| Park et al., 2010 | −3.67 [−6.26, −1.08] | 74.11 |
| Park et al., 2010 | −3.61 [−6.05, −1.17] | 74.24 |
| Pratiwi et al., 2019 | −3.25 [−5.58, −0.93] | 73.23 |
| Pratiwi et al., 2020 | −3.31 [−5.65, −0.96] | 73.58 |
| Song et al., 2019 | −3.72 [−6.17, −1.26] | 73.72 |
| Stigsdotter et al., 2017 | −3.57 [−6.04, −1.09] | 74.24 |
| Tsunetsugu et al., 2007 | −3.48 [−5.85, −1.12] | 74.21 |
| Tsunetsugu et al., 2007 | −3.60 [−5.97, −1.22] | 74.23 |
| Zheng et al., 2017 | −3.23 [−5.57, −0.88] | 72.52 |

Two trials from the same study are represented by the same study ID, the same below.

**Table S5. Sensitivity analysis of the effects of forest therapy and urban control on salivary cortisol concentration**

| Omitted study | Effect MD (95% CI) | Heterogeneity ($I^2$; %) |
|---|---|---|
| Aziz et al., 2021 | −3.13 [−5.82, −0.45] | 89.38 |
| Bang et al., 2016 | −3.39 [−6.00, −0.79] | 89.19 |
| Gong et al., 2017 | −3.54 [−6.11, −0.98] | 88.67 |
| Gong et al., 2017 | −3.54 [−6.10, −0.97] | 88.54 |
| Hassan et al., 2018 | −2.96 [−5.60, −0.32] | 89.14 |
| Janeczko et al., 2020 | −2.98 [−5.62, −0.35] | 89.24 |
| Kabisch et al., 2021 | −3.28 [−5.94, −0.63] | 89.29 |
| Kabisch et al., 2021 | −3.35 [−6.01, −0.70] | 89.09 |
| Kanelli et al., 2021 | −3.16 [−5.80, −0.51] | 89.38 |
| Lee and Lee, 2014 | −2.56 [−5.05, −0.07] | 88.00 |
| Lee et al., 2009 | −3.05 [−5.64, −0.45] | 89.36 |
| Lee et al., 2015 | −3.17 [−5.81, −0.54] | 89.38 |
| Lei et al., 2020 | −3.22 [−5.91, −0.53] | 89.35 |
| Li et al., 2021 | −3.38 [−5.99, −0.76] | 89.19 |
| Lyu et al., 2019 | −3.31 [−5.94, −0.68] | 89.28 |
| Mao et al., 2012 | −2.98 [−5.61, −0.35] | 89.27 |
| Park et al., 2010 | −3.24 [−6.10, −0.38] | 89.28 |
| Park et al., 2010 | −3.20 [−5.93, −0.46] | 89.37 |
| Pratiwi et al., 2019 | −2.85 [−5.45, −0.26] | 89.10 |
| Pratiwi et al., 2020 | −3.02 [−5.65, −0.39] | 89.32 |
| Song et al., 2019 | −3.24 [−5.95, −0.52] | 89.33 |
| Stigsdotter et al., 2017 | −3.16 [−5.86, −0.45] | 89.38 |
| Tsunetsugu et al., 2007 | −3.05 [−5.69, −0.40] | 89.33 |
| Tsunetsugu et al., 2007 | −3.16 [−5.79, −0.53] | 89.38 |
| Zheng et al., 2017 | −1.75 [−3.48, −0.03] | 73.99 |

**Table S6. Sensitivity analysis of the effects of forest therapy and urban control on systolic blood pressure**

| Omitted study | Effect MD (95% CI) | Heterogeneity ($I^2$; %) |
|---|---|---|
| Gidlow et al., 2016 | -0.07 [-0.10, -0.04] | 83.24 |
| Juong et al., 2020 | -0.07 [-0.10, -0.04] | 84.92 |
| Kanelli et al., 2021 | -0.06 [-0.09, -0.03] | 83.98 |
| Kobayashi et al., 2017 | -0.07 [-0.10, -0.03] | 84.7 |
| Komori et al., 2017 | -0.07 [-0.10, -0.04] | 84.92 |
| Lee et al., 2009 | -0.07 [-0.10, -0.04] | 84.83 |
| Lee et al., 2011 | -0.07 [-0.10, -0.04] | 84.92 |
| Lee et al., 2015 | -0.07 [-0.10, -0.04] | 84.88 |
| Park et al., 2007 | -0.05 [-0.06, -0.03] | 48.61 |
| Park et al., 2007 | -0.07 [-0.10, -0.04] | 83.66 |
| Park et al., 2008 | -0.07 [-0.10, -0.04] | 84.93 |
| Park et al., 2010 | -0.07 [-0.10, -0.04] | 84.87 |
| Park et al., 2010 | -0.07 [-0.10, -0.04] | 84.87 |
| Toda et al., 2013 | -0.07 [-0.10, -0.03] | 84.72 |
| Tsunetsugu et al., 2007 | -0.06 [-0.09, -0.03] | 84.54 |
| Tsunetsugu et al., 2007 | -0.06 [-0.09, -0.03] | 84.69 |

**Figure S1. Risk of bias graph for parallel randomized controlled trials studies**

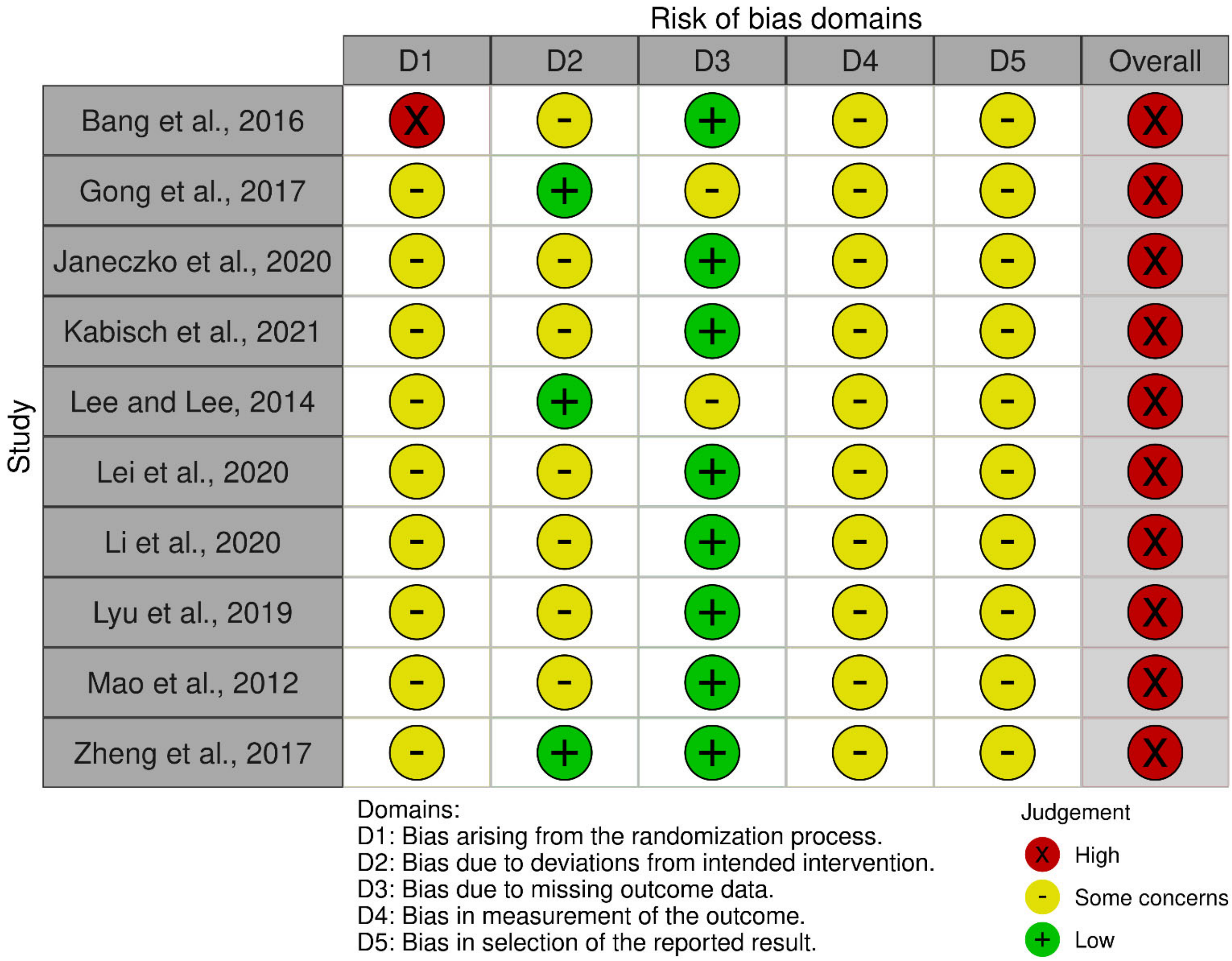

**Figure S2. Risk of bias graph for crossover randomized controlled trials studies**

| Study ID | D1 | DS | D2 | D3 | D4 | D5 | Overall |
|---|---|---|---|---|---|---|---|
| Abdul Aziz et al., 2021 | ! | ! | ! | + | ! | ! | - |
| Gidlow et al., 2016 | ! | ! | - | + | ! | ! | - |
| Hassan et al., 2018 | - | ! | - | + | ! | ! | - |
| Kobayashi et al., 2017 | - | ! | ! | + | ! | ! | - |
| Komori et al., 2017 | ! | ! | ! | + | ! | ! | - |
| Lee et al., 2009 | - | ! | ! | + | ! | ! | - |
| Lee et al., 2011 | - | ! | ! | + | ! | ! | - |
| Lee et al., 2015 | ! | ! | ! | + | ! | ! | - |
| Park et al., 2007 | ! | ! | ! | + | ! | ! | - |
| Park et al., 2008 | - | ! | ! | + | ! | ! | - |
| Park et al., 2010 | - | ! | - | - | ! | ! | - |
| Pratiwi et al., 2019 | - | ! | ! | + | ! | ! | - |
| Pratiwi et al., 2020 | ! | ! | + | + | ! | ! | - |
| Song et al., 2019 | ! | ! | ! | - | ! | ! | - |
| Tsunetsugu et al., 2007 | ! | ! | + | ! | ! | ! | - |

+ Low risk
! Some concerns
- High risk

D1 Randomisation process
DS Bias arising from period and carryover effects
D2 Deviations from the intended interventions
D3 Missing outcome data
D4 Measurement of the outcome
D5 Selection of the reported result

**Figure S3. Risk of bias graph for non-randomized controlled trials studies**

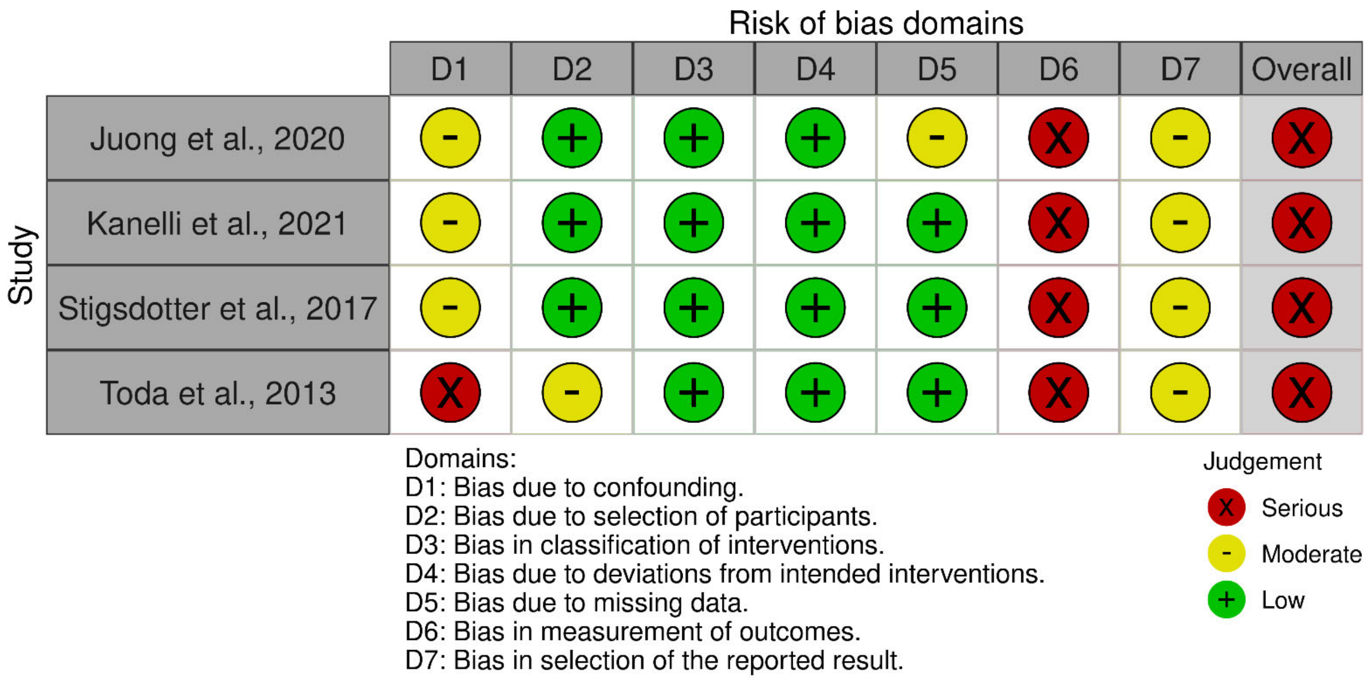

**Figure S4. Meta-analysis of the effects of forest therapy and urban control on systolic blood pressure**

| Study | Treatment N | Treatment Mean | Treatment SD | Control N | Control Mean | Control SD | Mean Difference with 95% CI | Weight (%) |
|---|---|---|---|---|---|---|---|---|
| Aziz et al., 2021 | 90 | 116.5 | 14.14 | 90 | 120.53 | 15.84 | -4.03 [ -8.42, 0.36] | 5.25 |
| Bang et al., 2016 | 18 | 123.5 | 16.97 | 27 | 115.89 | 10.87 | 7.61 [ -0.51, 15.73] | 3.59 |
| Gong et al., 2017 | 19 | 106.5 | 8.52 | 20 | 97.13 | 7.84 | 9.37 [ 4.24, 14.50] | 4.91 |
| Gong et al., 2017 | 19 | 105.89 | 8.66 | 20 | 99.03 | 9.64 | 6.86 [ 1.10, 12.62] | 4.61 |
| Hassan et al., 2018 | 60 | 110.41 | 12.01 | 60 | 118.01 | 12.55 | -7.60 [ -12.00, -3.20] | 5.25 |
| Janeczko et al., 2020 | 30 | 115.52 | 15.28 | 45 | 126.62 | 17.91 | -11.10 [ -18.91, -3.29] | 3.71 |
| Kabisch et al., 2021 | 66 | 134.27 | 17.71 | 33 | 135.79 | 17.72 | -1.52 [ -8.92, 5.88] | 3.88 |
| Kabisch et al., 2021 | 66 | 132.73 | 16.84 | 33 | 133.36 | 16.62 | -0.63 [ -7.64, 6.38] | 4.05 |
| Kanelli et al., 2021 | 24 | 117.6 | 12.25 | 24 | 120.2 | 13.72 | -2.60 [ -9.96, 4.76] | 3.90 |
| Lee and Lee, 2014 | 43 | 112.05 | 16.77 | 19 | 129.78 | 14.53 | -17.73 [ -26.44, -9.02] | 3.36 |
| Lee et al., 2015 | 11 | 114.1 | 11.28 | 11 | 122.6 | 11.28 | -8.50 [ -17.93, 0.93] | 3.11 |
| Lei et al., 2020 | 9 | 135.47 | 9.08 | 9 | 148.14 | 11.48 | -12.67 [ -22.23, -3.11] | 3.06 |
| Li et al., 2021 | 24 | 121.61 | 11.77 | 24 | 115.8 | 16.93 | 5.81 [ -2.44, 14.06] | 3.54 |
| Lyu et al., 2019 | 45 | 108.63 | 11.61 | 15 | 113.5 | 10.92 | -4.87 [ -11.56, 1.82] | 4.19 |
| Mao et al., 2012 | 12 | 137.83 | 14.38 | 12 | 148.33 | 10.18 | -10.50 [ -20.47, -0.53] | 2.93 |
| Park et al., 2010 | 268 | 119.09 | 13.25 | 268 | 121.1 | 13.45 | -2.01 [ -4.27, 0.25] | 6.11 |
| Park et al., 2010 | 75 | 126.62 | 13.04 | 75 | 128.76 | 14.5 | -2.14 [ -6.55, 2.27] | 5.24 |
| Pratiwi et al., 2019 | 24 | 122.85 | 14.74 | 24 | 133.99 | 17.63 | -11.14 [ -20.33, -1.95] | 3.19 |
| Pratiwi et al., 2020 | 36 | 126.2 | 19.19 | 36 | 135.7 | 19.86 | -9.50 [ -18.52, -0.48] | 3.25 |
| Song et al., 2019 | 65 | 96.3 | 10.48 | 65 | 96.8 | 9.67 | -0.50 [ -3.97, 2.97] | 5.66 |
| Stigsdotter et al., 2017 | 51 | 101.7 | 9.67 | 51 | 104.8 | 8.67 | -3.10 [ -6.66, 0.46] | 5.62 |
| Tsunetsugu et al., 2007 | 11 | 126.7 | 10.77 | 11 | 131.19 | 10.76 | -4.49 [ -13.49, 4.51] | 3.26 |
| Tsunetsugu et al., 2007 | 9 | 126.34 | 8.08 | 9 | 127.78 | 9.69 | -1.44 [ -9.68, 6.80] | 3.54 |
| Zheng et al., 2017 | 95 | 119 | 20 | 95 | 128 | 18 | -9.00 [ -14.41, -3.59] | 4.78 |
| **Overall** | | | | | | | -3.44 [ -5.74, -1.14] | |

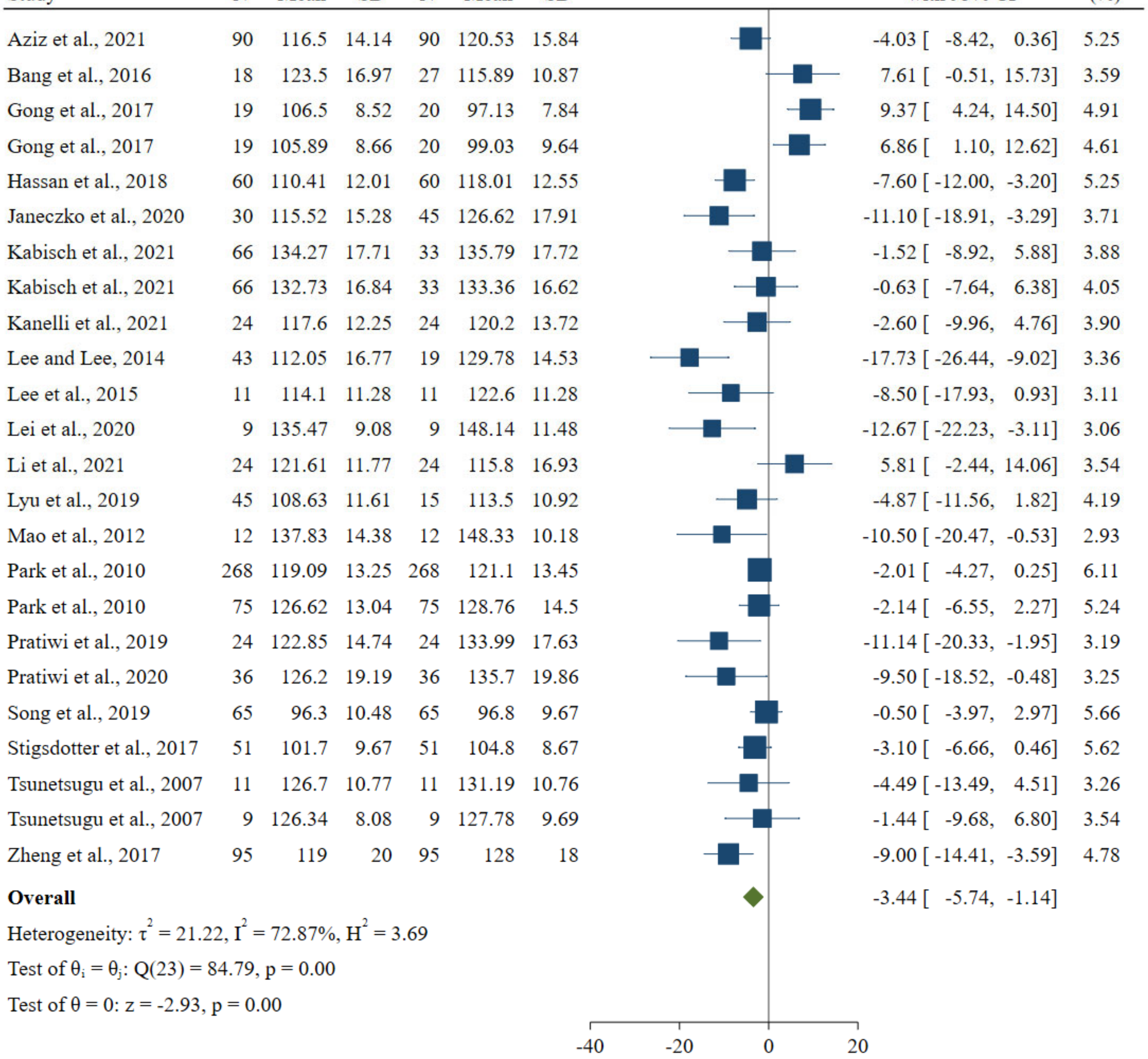


Heterogeneity: $\tau^2 = 21.22$, $I^2 = 72.87\%$, $H^2 = 3.69$

Test of $\theta_i = \theta_j$: $Q(23) = 84.79$, $p = 0.00$

Test of $\theta = 0$: $z = -2.93$, $p = 0.00$

Random-effects DerSimonian–Laird model

**Figure S5. Meta-analysis of the effects of forest therapy and urban control on diastolic blood pressure**

| Study | Treatment N | Treatment Mean | Treatment SD | Control N | Control Mean | Control SD | Mean Difference with 95% CI | Weight (%) |
|---|---|---|---|---|---|---|---|---|
| Aziz et al., 2021 | 90 | 70.3 | 14.89 | 90 | 73 | 10.06 | -2.70 [ -6.41, 1.01] | 4.34 |
| Bang et al., 2016 | 18 | 76.94 | 11.02 | 27 | 72.44 | 10.89 | 4.50 [ -2.03, 11.03] | 3.63 |
| Gong et al., 2017 | 19 | 67.89 | 9.5 | 20 | 60.33 | 6.66 | 7.56 [ 2.43, 12.69] | 4.00 |
| Gong et al., 2017 | 19 | 68 | 7.94 | 20 | 61.08 | 6.24 | 6.92 [ 2.45, 11.39] | 4.17 |
| Hassan et al., 2018 | 60 | 61.67 | 11 | 60 | 68.17 | 11.7 | -6.50 [ -10.56, -2.44] | 4.27 |
| Janeczko et al., 2020 | 30 | 69.84 | 9.73 | 45 | 75.95 | 11.03 | -6.11 [ -10.98, -1.24] | 4.07 |
| Kabisch et al., 2021 | 66 | 83.68 | 8.89 | 33 | 82.91 | 10.37 | 0.77 [ -3.16, 4.70] | 4.30 |
| Kabisch et al., 2021 | 66 | 80.79 | 8.65 | 33 | 78.55 | 8.6 | 2.24 [ -1.37, 5.85] | 4.37 |
| Kanelli et al., 2021 | 24 | 71.5 | 10.29 | 24 | 73.5 | 8.33 | -2.00 [ -7.30, 3.30] | 3.96 |
| Lee and Lee, 2014 | 43 | 66.58 | 13.7 | 19 | 83.11 | 8.94 | -16.53 [ -23.26, -9.80] | 3.58 |
| Lee et al., 2009 | 12 | 68.4 | 12.1 | 12 | 74.1 | 16.9 | -5.70 [ -17.46, 6.06] | 2.35 |
| Lee et al., 2015 | 11 | 58.5 | 7.3 | 11 | 60 | 6.96 | -1.50 [ -7.46, 4.46] | 3.79 |
| Lei et al., 2020 | 9 | 84.81 | 4.13 | 9 | 85.58 | 3.17 | -0.77 [ -4.17, 2.63] | 4.41 |
| Li et al., 2021 | 24 | 82.56 | 10.21 | 24 | 78.91 | 10.28 | 3.65 [ -2.15, 9.45] | 3.83 |
| Lyu et al., 2019 | 45 | 63.64 | 9.66 | 15 | 61.9 | 9.06 | 1.74 [ -3.82, 7.30] | 3.89 |
| Mao et al., 2012 | 12 | 80.84 | 6.97 | 12 | 87.19 | 7.28 | -6.35 [ -12.05, -0.65] | 3.85 |
| Park et al., 2010 | 268 | 59.89 | 10.71 | 268 | 60.79 | 10.13 | -0.90 [ -2.66, 0.86] | 4.68 |
| Park et al., 2010 | 75 | 66.41 | 8.31 | 75 | 67.86 | 8.84 | -1.45 [ -4.20, 1.30] | 4.54 |
| Pratiwi et al., 2019 | 24 | 68.53 | 12.8 | 24 | 78.32 | 9.84 | -9.79 [ -16.25, -3.33] | 3.65 |
| Pratiwi et al., 2020 | 36 | 72.83 | 12.7 | 36 | 78.23 | 13.01 | -5.40 [ -11.34, 0.54] | 3.79 |
| Song et al., 2019 | 65 | 54.9 | 8.87 | 65 | 55.5 | 8.06 | -0.60 [ -3.51, 2.31] | 4.51 |
| Stigsdotter et al., 2017 | 51 | 70.2 | 7.98 | 51 | 72.5 | 8.48 | -2.30 [ -5.50, 0.90] | 4.45 |
| Tsunetsugu et al., 2007 | 11 | 59.09 | 6.38 | 11 | 63.73 | 5.23 | -4.64 [ -9.52, 0.24] | 4.07 |
| Tsunetsugu et al., 2007 | 9 | 62.77 | 5.8 | 9 | 64.6 | 8.32 | -1.83 [ -8.46, 4.80] | 3.61 |
| Zheng et al., 2017 | 95 | 62 | 14 | 95 | 96 | 24 | -34.00 [ -39.59, -28.41] | 3.88 |
| **Overall** | | | | | | | -3.07 [ -5.59, -0.54] | |

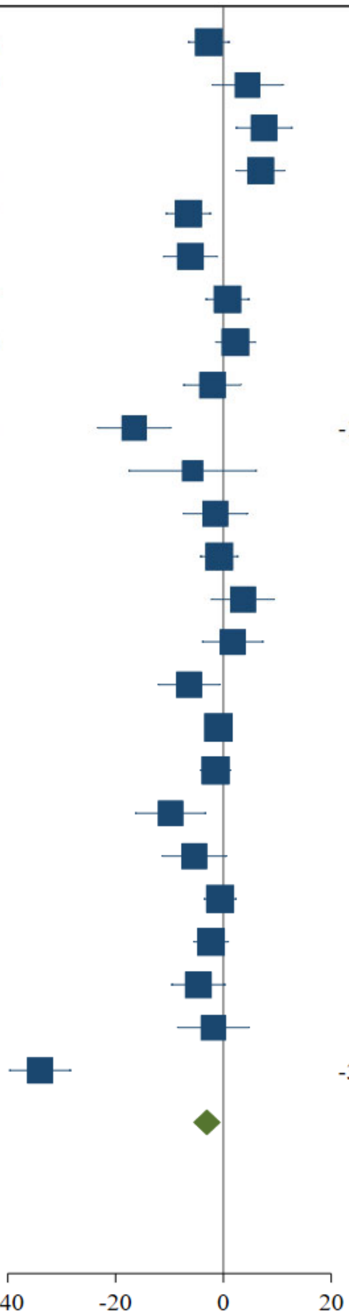


Heterogeneity: $\tau^2 = 34.72$, $I^2 = 88.59\%$, $H^2 = 8.76$

Test of $\theta_i = \theta_j$: $Q(24) = 210.26$, $p = 0.00$

Test of $\theta = 0$: $z = -2.38$, $p = 0.02$

Random-effects DerSimonian–Laird model

**Figure S6. Meta-analysis of the effects of forest therapy and urban control on salivary cortisol concentration**

| Study | Treatment N | Treatment Mean | Treatment SD | Control N | Control Mean | Control SD | Mean Difference with 95% CI | Weight (%) |
|---|---|---|---|---|---|---|---|---|
| Gidlow et al., 2016 | 37 | .14 | .08 | 37 | .14 | .06 | 0.00 [ -0.03, 0.03] | 7.75 |
| Juong et al., 2020 | 22 | .26 | .09 | 22 | .3 | .09 | -0.04 [ -0.09, 0.01] | 6.66 |
| Kanelli et al., 2021 | 24 | .1 | .05 | 24 | .2 | .1 | -0.10 [ -0.14, -0.06] | 7.12 |
| Kobayashi et al., 2017 | 348 | .25 | .1 | 348 | .29 | .12 | -0.04 [ -0.06, -0.02] | 8.34 |
| Komori et al., 2017 | 22 | .14 | .09 | 22 | .18 | .09 | -0.04 [ -0.09, 0.01] | 6.66 |
| Lee et al., 2009 | 12 | .24 | .04 | 12 | .27 | .07 | -0.03 [ -0.08, 0.02] | 7.08 |
| Lee et al., 2011 | 12 | .35 | .14 | 12 | .41 | .14 | -0.06 [ -0.17, 0.05] | 3.78 |
| Lee et al., 2015 | 11 | .22 | .07 | 11 | .29 | .12 | -0.07 [ -0.15, 0.01] | 5.09 |
| Park et al., 2007 | 9 | .33 | .1 | 9 | .69 | .06 | -0.36 [ -0.44, -0.28] | 5.40 |
| Park et al., 2007 | 12 | .6 | .09 | 12 | .55 | .09 | 0.05 [ -0.02, 0.12] | 5.62 |
| Park et al., 2008 | 10 | .27 | .08 | 10 | .32 | .1 | -0.05 [ -0.13, 0.03] | 5.23 |
| Park et al., 2010 | 260 | .3 | .14 | 260 | .34 | .17 | -0.04 [ -0.07, -0.01] | 7.99 |
| Park et al., 2010 | 74 | .3 | .11 | 74 | .36 | .14 | -0.06 [ -0.10, -0.02] | 7.34 |
| Toda et al., 2013 | 20 | .35 | .08 | 20 | .43 | .1 | -0.08 [ -0.14, -0.02] | 6.50 |
| Tsunetsugu et al., 2007 | 11 | .26 | .03 | 11 | .36 | .11 | -0.10 [ -0.17, -0.03] | 5.87 |
| Tsunetsugu et al., 2007 | 9 | .37 | .1 | 9 | .49 | .15 | -0.12 [ -0.24, -0.00] | 3.57 |
| **Overall** | | | | | | | -0.07 [ -0.10, -0.04] | |

Heterogeneity: $\tau^2 = 0.00$, $I^2 = 83.85\%$, $H^2 = 6.19$

Test of $\theta_i = \theta_j$: $Q(15) = 92.91$, $p = 0.00$

Test of $\theta = 0$: $z = -4.44$, $p = 0.00$

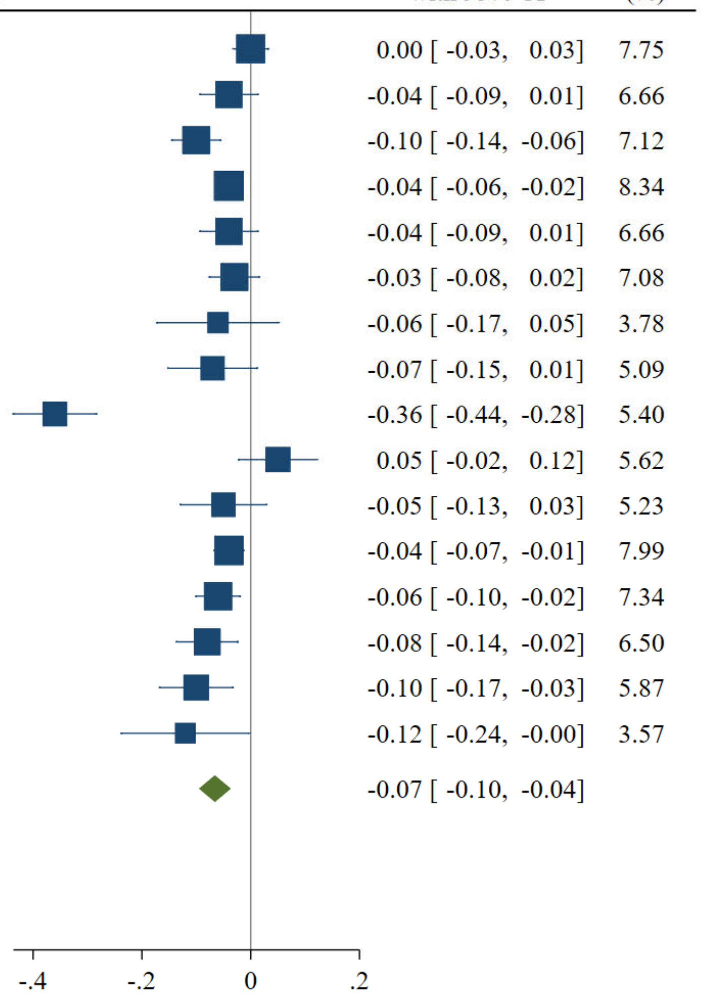


Random-effects DerSimonian–Laird model

**Figure S7. Study design-based subgroup analysis of the effects of forest therapy and urban control on systolic blood pressure**

| | Treatment | | | Control | | | | |
|---|---|---|---|---|---|---|---|---|
| Subgroup | N | Mean | SD | N | Mean | SD | Mean difference with 95% CI | Weight (%) |
| **RCT** | | | | | | | | |
| Aziz et al., 2021 | 90 | 116.5 | 14.14 | 90 | 120.53 | 15.84 | -4.03 [ -8.42, 0.36] | 5.25 |
| Bang et al., 2016 | 18 | 123.5 | 16.97 | 27 | 115.89 | 10.87 | 7.61 [ -0.51, 15.73] | 3.59 |
| Gong et al., 2017 | 19 | 106.5 | 8.52 | 20 | 97.13 | 7.84 | 9.37 [ 4.24, 14.50] | 4.91 |
| Gong et al., 2017 | 19 | 105.89 | 8.66 | 20 | 99.03 | 9.64 | 6.86 [ 1.10, 12.62] | 4.61 |
| Hassan et al., 2018 | 60 | 110.41 | 12.01 | 60 | 118.01 | 12.55 | -7.60 [-12.00, -3.20] | 5.25 |
| Janeczko et al., 2020 | 30 | 115.52 | 15.28 | 45 | 126.62 | 17.91 | -11.10 [-18.91, -3.29] | 3.71 |
| Kabisch et al., 2021 | 66 | 134.27 | 17.71 | 33 | 135.79 | 17.72 | -1.52 [ -8.92, 5.88] | 3.88 |
| Kabisch et al., 2021 | 66 | 132.73 | 16.84 | 33 | 133.36 | 16.62 | -0.63 [ -7.64, 6.38] | 4.05 |
| Lee and Lee, 2014 | 43 | 112.05 | 16.77 | 19 | 129.78 | 14.53 | -17.73 [-26.44, -9.02] | 3.36 |
| Lee et al., 2015 | 11 | 114.1 | 11.28 | 11 | 122.6 | 11.28 | -8.50 [-17.93, 0.93] | 3.11 |
| Lei et al., 2020 | 9 | 135.47 | 9.08 | 9 | 148.14 | 11.48 | -12.67 [-22.23, -3.11] | 3.06 |
| Li et al., 2021 | 24 | 121.61 | 11.77 | 24 | 115.8 | 16.93 | 5.81 [ -2.44, 14.06] | 3.54 |
| Lyu et al., 2019 | 45 | 108.63 | 11.61 | 15 | 113.5 | 10.92 | -4.87 [-11.56, 1.82] | 4.19 |
| Mao et al., 2012 | 12 | 137.83 | 14.38 | 12 | 148.33 | 10.18 | -10.50 [-20.47, -0.53] | 2.93 |
| Park et al., 2010 | 268 | 119.09 | 13.25 | 268 | 121.1 | 13.45 | -2.01 [ -4.27, 0.25] | 6.11 |
| Park et al., 2010 | 75 | 126.62 | 13.04 | 75 | 128.76 | 14.5 | -2.14 [ -6.55, 2.27] | 5.24 |
| Pratiwi et al., 2019 | 24 | 122.85 | 14.74 | 24 | 133.99 | 17.63 | -11.14 [-20.33, -1.95] | 3.19 |
| Pratiwi et al., 2020 | 36 | 126.2 | 19.19 | 36 | 135.7 | 19.86 | -9.50 [-18.52, -0.48] | 3.25 |
| Song et al., 2019 | 65 | 96.3 | 10.48 | 65 | 96.8 | 9.67 | -0.50 [ -3.97, 2.97] | 5.66 |
| Tsunetsugu et al., 2007 | 11 | 126.7 | 10.77 | 11 | 131.19 | 10.76 | -4.49 [-13.49, 4.51] | 3.26 |
| Tsunetsugu et al., 2007 | 9 | 126.34 | 8.08 | 9 | 127.78 | 9.69 | -1.44 [ -9.68, 6.80] | 3.54 |
| Zheng et al., 2017 | 95 | 119 | 20 | 95 | 128 | 18 | -9.00 [-14.41, -3.59] | 4.78 |
| Heterogeneity: $\tau^2 = 25.17$, $I^2 = 75.21\%$, $H^2 = 4.03$ | | | | | | | -3.55 [ -6.12, -0.99] | |
| Test of $\theta_i = \theta_j$: Q(21) = 84.72, p = 0.00 | | | | | | | | |
| **Non-RCT** | | | | | | | | |
| Kanelli et al., 2021 | 24 | 117.6 | 12.25 | 24 | 120.2 | 13.72 | -2.60 [ -9.96, 4.76] | 3.90 |
| Stigsdotter et al., 2017 | 51 | 101.7 | 9.67 | 51 | 104.8 | 8.67 | -3.10 [ -6.66, 0.46] | 5.62 |
| Heterogeneity: $\tau^2 = 0.00$, $I^2 = 0.00\%$, $H^2 = 1.00$ | | | | | | | -3.00 [ -6.21, 0.20] | |
| Test of $\theta_i = \theta_j$: Q(1) = 0.01, p = 0.90 | | | | | | | | |
| **Overall** | | | | | | | -3.44 [ -5.74, -1.14] | |
| Heterogeneity: $\tau^2 = 21.22$, $I^2 = 72.87\%$, $H^2 = 3.69$ | | | | | | | | |
| Test of $\theta_i = \theta_j$: Q(23) = 84.79, p = 0.00 | | | | | | | | |
| Test of group differences: $Q_b(1) = 0.07$, p = 0.79 | | | | | | | | |

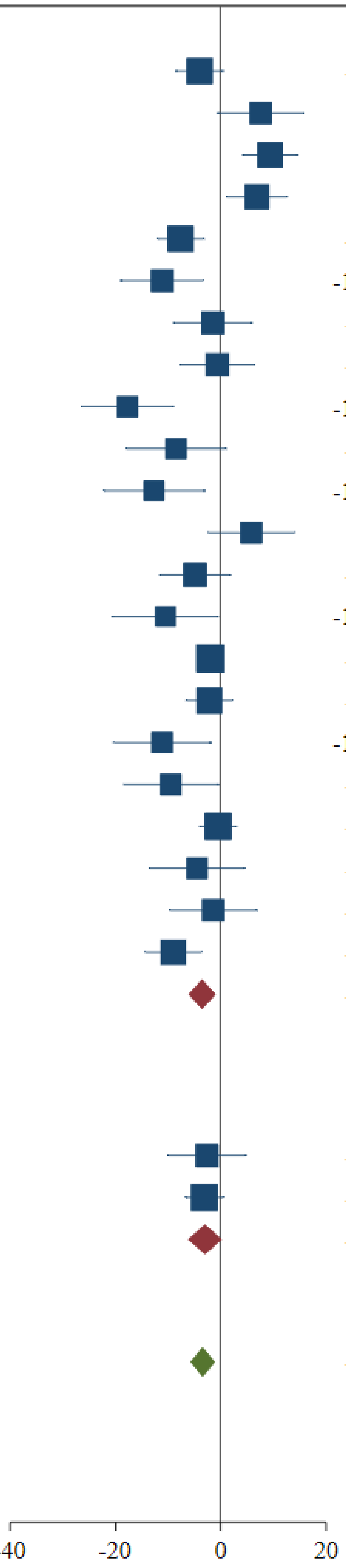


Random-effects DerSimonian–Laird model

**Figure S8. Session-based subgroup analysis of the effects of forest therapy and urban control on systolic blood pressure**

| Subgroup | Treatment N | Treatment Mean | Treatment SD | Control N | Control Mean | Control SD | Mean difference with 95% CI | Weight (%) |
|---|---|---|---|---|---|---|---|---|
| **Seated viewing** | | | | | | | | |
| Gong et al., 2017 | 19 | 105.89 | 8.66 | 20 | 99.03 | 9.64 | 6.86 [ 1.10, 12.62] | 4.61 |
| Kabisch et al., 2021 | 66 | 134.27 | 17.71 | 33 | 135.79 | 17.72 | -1.52 [ -8.92, 5.88] | 3.88 |
| Lee et al., 2015 | 11 | 114.1 | 11.28 | 11 | 122.6 | 11.28 | -8.50 [ -17.93, 0.93] | 3.11 |
| Park et al., 2010 | 268 | 119.09 | 13.25 | 268 | 121.1 | 13.45 | -2.01 [ -4.27, 0.25] | 6.11 |
| Pratiwi et al., 2019 | 24 | 122.85 | 14.74 | 24 | 133.99 | 17.63 | -11.14 [ -20.33, -1.95] | 3.19 |
| Song et al., 2019 | 65 | 96.3 | 10.48 | 65 | 96.8 | 9.67 | -0.50 [ -3.97, 2.97] | 5.66 |
| Tsunetsugu et al., 2007 | 11 | 126.7 | 10.77 | 11 | 131.19 | 10.76 | -4.49 [ -13.49, 4.51] | 3.26 |
| Heterogeneity: $\tau^2 = 10.44$, $I^2 = 60.96\%$, $H^2 = 2.56$ | | | | | | | -1.89 [ -5.23, 1.46] | |
| Test of $\theta_i = \theta_j$: $Q(6) = 15.37$, $p = 0.02$ | | | | | | | | |
| **Walking or multi-session program** | | | | | | | | |
| Aziz et al., 2021 | 90 | 116.5 | 14.14 | 90 | 120.53 | 15.84 | -4.03 [ -8.42, 0.36] | 5.25 |
| Bang et al., 2016 | 18 | 123.5 | 16.97 | 27 | 115.89 | 10.87 | 7.61 [ -0.51, 15.73] | 3.59 |
| Gong et al., 2017 | 19 | 106.5 | 8.52 | 20 | 97.13 | 7.84 | 9.37 [ 4.24, 14.50] | 4.91 |
| Hassan et al., 2018 | 60 | 110.41 | 12.01 | 60 | 118.01 | 12.55 | -7.60 [ -12.00, -3.20] | 5.25 |
| Janeczko et al., 2020 | 30 | 115.52 | 15.28 | 45 | 126.62 | 17.91 | -11.10 [ -18.91, -3.29] | 3.71 |
| Kabisch et al., 2021 | 66 | 132.73 | 16.84 | 33 | 133.36 | 16.62 | -0.63 [ -7.64, 6.38] | 4.05 |
| Kanelli et al., 2021 | 24 | 117.6 | 12.25 | 24 | 120.2 | 13.72 | -2.60 [ -9.96, 4.76] | 3.90 |
| Lee and Lee, 2014 | 43 | 112.05 | 16.77 | 19 | 129.78 | 14.53 | -17.73 [ -26.44, -9.02] | 3.36 |
| Lei et al., 2020 | 9 | 135.47 | 9.08 | 9 | 148.14 | 11.48 | -12.67 [ -22.23, -3.11] | 3.06 |
| Li et al., 2021 | 24 | 121.61 | 11.77 | 24 | 115.8 | 16.93 | 5.81 [ -2.44, 14.06] | 3.54 |
| Lyu et al., 2019 | 45 | 108.63 | 11.61 | 15 | 113.5 | 10.92 | -4.87 [ -11.56, 1.82] | 4.19 |
| Mao et al., 2012 | 12 | 137.83 | 14.38 | 12 | 148.33 | 10.18 | -10.50 [ -20.47, -0.53] | 2.93 |
| Park et al., 2010 | 75 | 126.62 | 13.04 | 75 | 128.76 | 14.5 | -2.14 [ -6.55, 2.27] | 5.24 |
| Pratiwi et al., 2020 | 36 | 126.2 | 19.19 | 36 | 135.7 | 19.86 | -9.50 [ -18.52, -0.48] | 3.25 |
| Stigsdotter et al., 2017 | 51 | 101.7 | 9.67 | 51 | 104.8 | 8.67 | -3.10 [ -6.66, 0.46] | 5.62 |
| Tsunetsugu et al., 2007 | 9 | 126.34 | 8.08 | 9 | 127.78 | 9.69 | -1.44 [ -9.68, 6.80] | 3.54 |
| Zheng et al., 2017 | 95 | 119 | 20 | 95 | 128 | 18 | -9.00 [ -14.41, -3.59] | 4.78 |
| Heterogeneity: $\tau^2 = 30.11$, $I^2 = 75.80\%$, $H^2 = 4.13$ | | | | | | | -4.04 [ -7.14, -0.94] | |
| Test of $\theta_i = \theta_j$: $Q(16) = 66.12$, $p = 0.00$ | | | | | | | | |
| **Overall** | | | | | | | -3.44 [ -5.74, -1.14] | |
| Heterogeneity: $\tau^2 = 21.22$, $I^2 = 72.87\%$, $H^2 = 3.69$ | | | | | | | | |
| Test of $\theta_i = \theta_j$: $Q(23) = 84.79$, $p = 0.00$ | | | | | | | | |
| Test of group differences: $Q_b(1) = 0.85$, $p = 0.36$ | | | | | | | | |

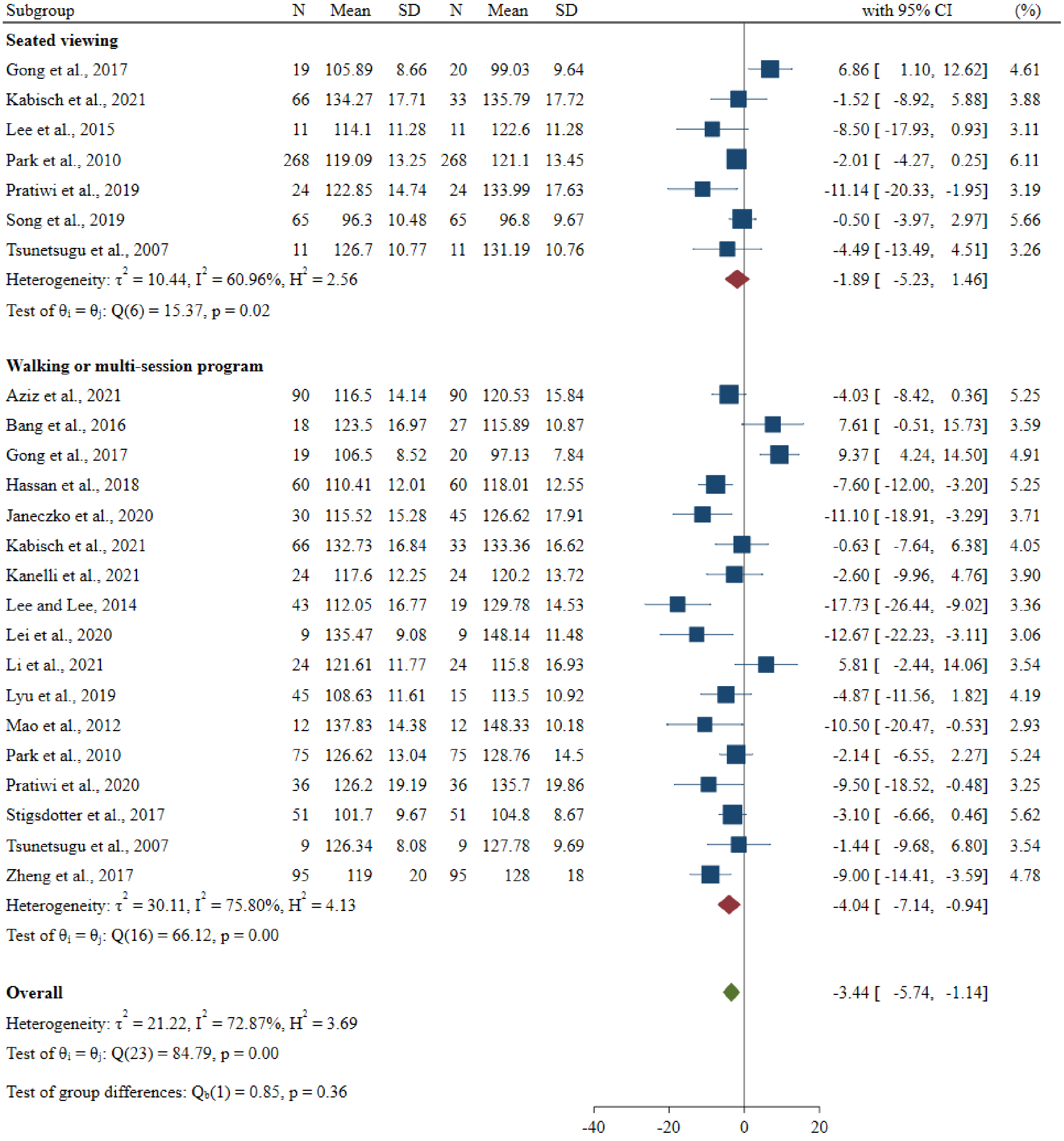


Random-effects DerSimonian–Laird model

**Figure S9. Duration-based subgroup analysis of the effects of forest therapy and urban control on systolic blood pressure**

| Subgroup | Treatment N | Treatment Mean | Treatment SD | Control N | Control Mean | Control SD | Mean difference with 95% CI | Weight (%) |
|---|---|---|---|---|---|---|---|---|
| **< 20 minutes** | | | | | | | | |
| Hassan et al., 2018 | 60 | 110.41 | 12.01 | 60 | 118.01 | 12.55 | -7.60 [ -12.00, -3.20] | 5.25 |
| Kabisch et al., 2021 | 66 | 134.27 | 17.71 | 33 | 135.79 | 17.72 | -1.52 [ -8.92, 5.88] | 3.88 |
| Kabisch et al., 2021 | 66 | 132.73 | 16.84 | 33 | 133.36 | 16.62 | -0.63 [ -7.64, 6.38] | 4.05 |
| Lee et al., 2015 | 11 | 114.1 | 11.28 | 11 | 122.6 | 11.28 | -8.50 [ -17.93, 0.93] | 3.11 |
| Li et al., 2021 | 24 | 121.61 | 11.77 | 24 | 115.8 | 16.93 | 5.81 [ -2.44, 14.06] | 3.54 |
| Park et al., 2010 | 268 | 119.09 | 13.25 | 268 | 121.1 | 13.45 | -2.01 [ -4.27, 0.25] | 6.11 |
| Park et al., 2010 | 75 | 126.62 | 13.04 | 75 | 128.76 | 14.5 | -2.14 [ -6.55, 2.27] | 5.24 |
| Pratiwi et al., 2019 | 24 | 122.85 | 14.74 | 24 | 133.99 | 17.63 | -11.14 [ -20.33, -1.95] | 3.19 |
| Song et al., 2019 | 65 | 96.3 | 10.48 | 65 | 96.8 | 9.67 | -0.50 [ -3.97, 2.97] | 5.66 |
| Tsunetsugu et al., 2007 | 11 | 126.7 | 10.77 | 11 | 131.19 | 10.76 | -4.49 [ -13.49, 4.51] | 3.26 |
| Tsunetsugu et al., 2007 | 9 | 126.34 | 8.08 | 9 | 127.78 | 9.69 | -1.44 [ -9.68, 6.80] | 3.54 |
| Heterogeneity: $\tau^2 = 4.20$, $I^2 = 37.87\%$, $H^2 = 1.61$ | | | | | | | -2.72 [ -4.84, -0.60] | |
| Test of $\theta_i = \theta_j$: Q(10) = 16.09, p = 0.10 | | | | | | | | |
| **≥ 20 minutes** | | | | | | | | |
| Aziz et al., 2021 | 90 | 116.5 | 14.14 | 90 | 120.53 | 15.84 | -4.03 [ -8.42, 0.36] | 5.25 |
| Bang et al., 2016 | 18 | 123.5 | 16.97 | 27 | 115.89 | 10.87 | 7.61 [ -0.51, 15.73] | 3.59 |
| Gong et al., 2017 | 19 | 106.5 | 8.52 | 20 | 97.13 | 7.84 | 9.37 [ 4.24, 14.50] | 4.91 |
| Gong et al., 2017 | 19 | 105.89 | 8.66 | 20 | 99.03 | 9.64 | 6.86 [ 1.10, 12.62] | 4.61 |
| Janeczko et al., 2020 | 30 | 115.52 | 15.28 | 45 | 126.62 | 17.91 | -11.10 [ -18.91, -3.29] | 3.71 |
| Kanelli et al., 2021 | 24 | 117.6 | 12.25 | 24 | 120.2 | 13.72 | -2.60 [ -9.96, 4.76] | 3.90 |
| Lee and Lee, 2014 | 43 | 112.05 | 16.77 | 19 | 129.78 | 14.53 | -17.73 [ -26.44, -9.02] | 3.36 |
| Lei et al., 2020 | 9 | 135.47 | 9.08 | 9 | 148.14 | 11.48 | -12.67 [ -22.23, -3.11] | 3.06 |
| Lyu et al., 2019 | 45 | 108.63 | 11.61 | 15 | 113.5 | 10.92 | -4.87 [ -11.56, 1.82] | 4.19 |
| Mao et al., 2012 | 12 | 137.83 | 14.38 | 12 | 148.33 | 10.18 | -10.50 [ -20.47, -0.53] | 2.93 |
| Pratiwi et al., 2020 | 36 | 126.2 | 19.19 | 36 | 135.7 | 19.86 | -9.50 [ -18.52, -0.48] | 3.25 |
| Stigsdotter et al., 2017 | 51 | 101.7 | 9.67 | 51 | 104.8 | 8.67 | -3.10 [ -6.66, 0.46] | 5.62 |
| Zheng et al., 2017 | 95 | 119 | 20 | 95 | 128 | 18 | -9.00 [ -14.41, -3.59] | 4.78 |
| Heterogeneity: $\tau^2 = 47.32$, $I^2 = 82.49\%$, $H^2 = 5.71$ | | | | | | | -4.25 [ -8.47, -0.02] | |
| Test of $\theta_i = \theta_j$: Q(12) = 68.55, p = 0.00 | | | | | | | | |
| **Overall** | | | | | | | -3.44 [ -5.74, -1.14] | |
| Heterogeneity: $\tau^2 = 21.22$, $I^2 = 72.87\%$, $H^2 = 3.69$ | | | | | | | | |
| Test of $\theta_i = \theta_j$: Q(23) = 84.79, p = 0.00 | | | | | | | | |
| Test of group differences: $Q_b(1) = 0.40$, p = 0.53 | | | | | | | | |

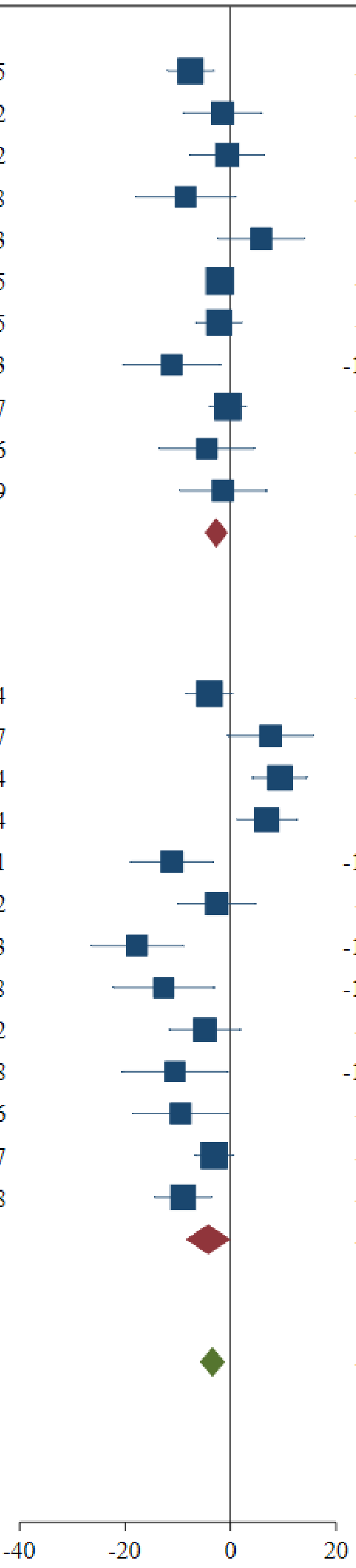


Random-effects DerSimonian–Laird model

**Figure S10. Study design-based subgroup analysis of the effects of forest therapy and urban control on diastolic blood pressure**

| Subgroup | Treatment N | Treatment Mean | Treatment SD | Control N | Control Mean | Control SD | Mean difference with 95% CI | Weight (%) |
|---|---|---|---|---|---|---|---|---|
| **RCT** | | | | | | | | |
| Aziz et al., 2021 | 90 | 70.3 | 14.89 | 90 | 73 | 10.06 | -2.70 [ -6.41, 1.01] | 4.34 |
| Bang et al., 2016 | 18 | 76.94 | 11.02 | 27 | 72.44 | 10.89 | 4.50 [ -2.03, 11.03] | 3.63 |
| Gong et al., 2017 | 19 | 67.89 | 9.5 | 20 | 60.33 | 6.66 | 7.56 [ 2.43, 12.69] | 4.00 |
| Gong et al., 2017 | 19 | 68 | 7.94 | 20 | 61.08 | 6.24 | 6.92 [ 2.45, 11.39] | 4.17 |
| Hassan et al., 2018 | 60 | 61.67 | 11 | 60 | 68.17 | 11.7 | -6.50 [ -10.56, -2.44] | 4.27 |
| Janeczko et al., 2020 | 30 | 69.84 | 9.73 | 45 | 75.95 | 11.03 | -6.11 [ -10.98, -1.24] | 4.07 |
| Kabisch et al., 2021 | 66 | 83.68 | 8.89 | 33 | 82.91 | 10.37 | 0.77 [ -3.16, 4.70] | 4.30 |
| Kabisch et al., 2021 | 66 | 80.79 | 8.65 | 33 | 78.55 | 8.6 | 2.24 [ -1.37, 5.85] | 4.37 |
| Lee and Lee, 2014 | 43 | 66.58 | 13.7 | 19 | 83.11 | 8.94 | -16.53 [ -23.26, -9.80] | 3.58 |
| Lee et al., 2009 | 12 | 68.4 | 12.1 | 12 | 74.1 | 16.9 | -5.70 [ -17.46, 6.06] | 2.35 |
| Lee et al., 2015 | 11 | 58.5 | 7.3 | 11 | 60 | 6.96 | -1.50 [ -7.46, 4.46] | 3.79 |
| Lei et al., 2020 | 9 | 84.81 | 4.13 | 9 | 85.58 | 3.17 | -0.77 [ -4.17, 2.63] | 4.41 |
| Li et al., 2021 | 24 | 82.56 | 10.21 | 24 | 78.91 | 10.28 | 3.65 [ -2.15, 9.45] | 3.83 |
| Lyu et al., 2019 | 45 | 63.64 | 9.66 | 15 | 61.9 | 9.06 | 1.74 [ -3.82, 7.30] | 3.89 |
| Mao et al., 2012 | 12 | 80.84 | 6.97 | 12 | 87.19 | 7.28 | -6.35 [ -12.05, -0.65] | 3.85 |
| Park et al., 2010 | 268 | 59.89 | 10.71 | 268 | 60.79 | 10.13 | -0.90 [ -2.66, 0.86] | 4.68 |
| Park et al., 2010 | 75 | 66.41 | 8.31 | 75 | 67.86 | 8.84 | -1.45 [ -4.20, 1.30] | 4.54 |
| Pratiwi et al., 2019 | 24 | 68.53 | 12.8 | 24 | 78.32 | 9.84 | -9.79 [ -16.25, -3.33] | 3.65 |
| Pratiwi et al., 2020 | 36 | 72.83 | 12.7 | 36 | 78.23 | 13.01 | -5.40 [ -11.34, 0.54] | 3.79 |
| Song et al., 2019 | 65 | 54.9 | 8.87 | 65 | 55.5 | 8.06 | -0.60 [ -3.51, 2.31] | 4.51 |
| Tsunetsugu et al., 2007 | 11 | 59.09 | 6.38 | 11 | 63.73 | 5.23 | -4.64 [ -9.52, 0.24] | 4.07 |
| Tsunetsugu et al., 2007 | 9 | 62.77 | 5.8 | 9 | 64.6 | 8.32 | -1.83 [ -8.46, 4.80] | 3.61 |
| Zheng et al., 2017 | 95 | 62 | 14 | 95 | 96 | 24 | -34.00 [ -39.59, -28.41] | 3.88 |
| Heterogeneity: $\tau^2 = 38.92$, $I^2 = 89.53\%$, $H^2 = 9.56$ | | | | | | | -3.17 [ -5.94, -0.39] | |
| Test of $\theta_i = \theta_j$: Q(22) = 210.21, p = 0.00 | | | | | | | | |
| **Non-RCT** | | | | | | | | |
| Kanelli et al., 2021 | 24 | 71.5 | 10.29 | 24 | 73.5 | 8.33 | -2.00 [ -7.30, 3.30] | 3.96 |
| Stigsdotter et al., 2017 | 51 | 70.2 | 7.98 | 51 | 72.5 | 8.48 | -2.30 [ -5.50, 0.90] | 4.45 |
| Heterogeneity: $\tau^2 = 0.00$, $I^2 = 0.00\%$, $H^2 = 1.00$ | | | | | | | -2.22 [ -4.96, 0.52] | |
| Test of $\theta_i = \theta_j$: Q(1) = 0.01, p = 0.92 | | | | | | | | |
| **Overall** | | | | | | | -3.07 [ -5.59, -0.54] | |
| Heterogeneity: $\tau^2 = 34.72$, $I^2 = 88.59\%$, $H^2 = 8.76$ | | | | | | | | |
| Test of $\theta_i = \theta_j$: Q(24) = 210.26, p = 0.00 | | | | | | | | |
| Test of group differences: $Q_b(1) = 0.23$, p = 0.63 | | | | | | | | |

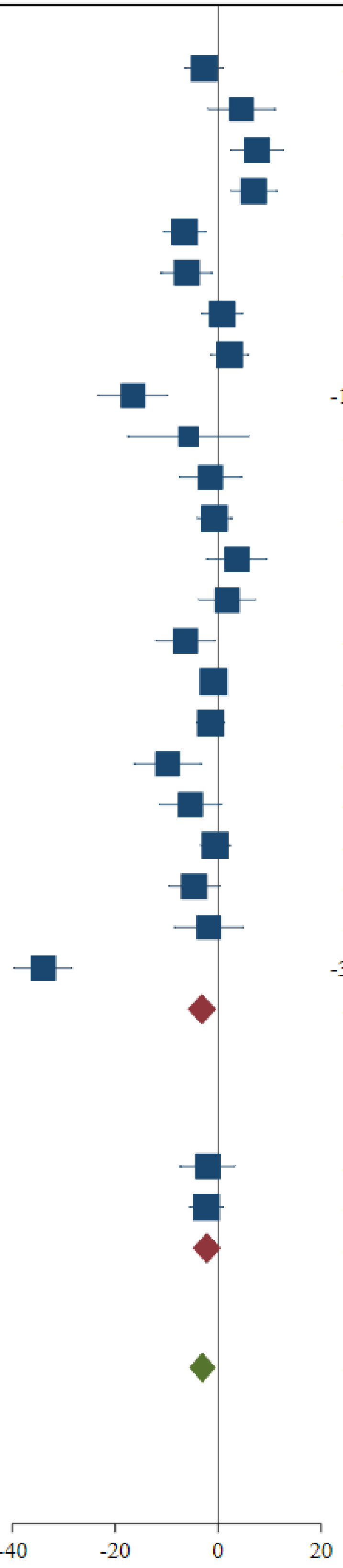


Random-effects DerSimonian–Laird model

**Figure S11. Session-based subgroup analysis of the effects of forest therapy and urban control on diastolic blood pressure**

| Subgroup | Treatment N | Treatment Mean | Treatment SD | Control N | Control Mean | Control SD | Mean difference with 95% CI | Weight (%) |
|---|---|---|---|---|---|---|---|---|
| **Seated viewing** | | | | | | | | |
| Gong et al., 2017 | 19 | 68 | 7.94 | 20 | 61.08 | 6.24 | 6.92 [ 2.45, 11.39] | 4.17 |
| Kabisch et al., 2021 | 66 | 83.68 | 8.89 | 33 | 82.91 | 10.37 | 0.77 [ -3.16, 4.70] | 4.30 |
| Lee et al., 2009 | 12 | 68.4 | 12.1 | 12 | 74.1 | 16.9 | -5.70 [ -17.46, 6.06] | 2.35 |
| Lee et al., 2015 | 11 | 58.5 | 7.3 | 11 | 60 | 6.96 | -1.50 [ -7.46, 4.46] | 3.79 |
| Park et al., 2010 | 268 | 59.89 | 10.71 | 268 | 60.79 | 10.13 | -0.90 [ -2.66, 0.86] | 4.68 |
| Pratiwi et al., 2019 | 24 | 68.53 | 12.8 | 24 | 78.32 | 9.84 | -9.79 [ -16.25, -3.33] | 3.65 |
| Song et al., 2019 | 65 | 54.9 | 8.87 | 65 | 55.5 | 8.06 | -0.60 [ -3.51, 2.31] | 4.51 |
| Tsunetsugu et al., 2007 | 11 | 59.09 | 6.38 | 11 | 63.73 | 5.23 | -4.64 [ -9.52, 0.24] | 4.07 |
| Heterogeneity: $\tau^2 = 8.82$, $I^2 = 69.08\%$, $H^2 = 3.23$ | | | | | | | -1.13 [ -3.80, 1.54] | |
| Test of $\theta_i = \theta_j$: $Q(7) = 22.64$, $p = 0.00$ | | | | | | | | |
| **Walking or multi-session program** | | | | | | | | |
| Aziz et al., 2021 | 90 | 70.3 | 14.89 | 90 | 73 | 10.06 | -2.70 [ -6.41, 1.01] | 4.34 |
| Bang et al., 2016 | 18 | 76.94 | 11.02 | 27 | 72.44 | 10.89 | 4.50 [ -2.03, 11.03] | 3.63 |
| Gong et al., 2017 | 19 | 67.89 | 9.5 | 20 | 60.33 | 6.66 | 7.56 [ 2.43, 12.69] | 4.00 |
| Hassan et al., 2018 | 60 | 61.67 | 11 | 60 | 68.17 | 11.7 | -6.50 [ -10.56, -2.44] | 4.27 |
| Janeczko et al., 2020 | 30 | 69.84 | 9.73 | 45 | 75.95 | 11.03 | -6.11 [ -10.98, -1.24] | 4.07 |
| Kabisch et al., 2021 | 66 | 80.79 | 8.65 | 33 | 78.55 | 8.6 | 2.24 [ -1.37, 5.85] | 4.37 |
| Kanelli et al., 2021 | 24 | 71.5 | 10.29 | 24 | 73.5 | 8.33 | -2.00 [ -7.30, 3.30] | 3.96 |
| Lee and Lee, 2014 | 43 | 66.58 | 13.7 | 19 | 83.11 | 8.94 | -16.53 [ -23.26, -9.80] | 3.58 |
| Lei et al., 2020 | 9 | 84.81 | 4.13 | 9 | 85.58 | 3.17 | -0.77 [ -4.17, 2.63] | 4.41 |
| Li et al., 2021 | 24 | 82.56 | 10.21 | 24 | 78.91 | 10.28 | 3.65 [ -2.15, 9.45] | 3.83 |
| Lyu et al., 2019 | 45 | 63.64 | 9.66 | 15 | 61.9 | 9.06 | 1.74 [ -3.82, 7.30] | 3.89 |
| Mao et al., 2012 | 12 | 80.84 | 6.97 | 12 | 87.19 | 7.28 | -6.35 [ -12.05, -0.65] | 3.85 |
| Park et al., 2010 | 75 | 66.41 | 8.31 | 75 | 67.86 | 8.84 | -1.45 [ -4.20, 1.30] | 4.54 |
| Pratiwi et al., 2020 | 36 | 72.83 | 12.7 | 36 | 78.23 | 13.01 | -5.40 [ -11.34, 0.54] | 3.79 |
| Stigsdotter et al., 2017 | 51 | 70.2 | 7.98 | 51 | 72.5 | 8.48 | -2.30 [ -5.50, 0.90] | 4.45 |
| Tsunetsugu et al., 2007 | 9 | 62.77 | 5.8 | 9 | 64.6 | 8.32 | -1.83 [ -8.46, 4.80] | 3.61 |
| Zheng et al., 2017 | 95 | 62 | 14 | 95 | 96 | 24 | -34.00 [ -39.59, -28.41] | 3.88 |
| Heterogeneity: $\tau^2 = 53.98$, $I^2 = 91.15\%$, $H^2 = 11.30$ | | | | | | | -3.81 [ -7.51, -0.11] | |
| Test of $\theta_i = \theta_j$: $Q(16) = 180.79$, $p = 0.00$ | | | | | | | | |
| **Overall** | | | | | | | -3.07 [ -5.59, -0.54] | |
| Heterogeneity: $\tau^2 = 34.72$, $I^2 = 88.59\%$, $H^2 = 8.76$ | | | | | | | | |
| Test of $\theta_i = \theta_j$: $Q(24) = 210.26$, $p = 0.00$ | | | | | | | | |
| Test of group differences: $Q_b(1) = 1.32$, $p = 0.25$ | | | | | | | | |

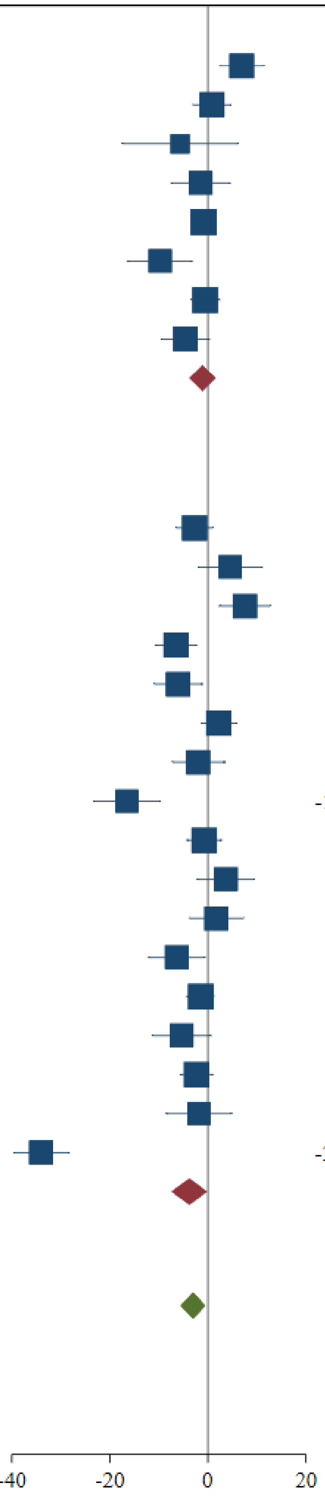


Random-effects DerSimonian–Laird model

**Figure S12. Duration-based subgroup analysis of the effects of forest therapy and urban control on diastolic blood pressure**

| Subgroup | Treatment N | Treatment Mean | Treatment SD | Control N | Control Mean | Control SD | Mean difference with 95% CI | Weight (%) |
|---|---|---|---|---|---|---|---|---|
| **< 20 minutes** | | | | | | | | |
| Hassan et al., 2018 | 60 | 61.67 | 11 | 60 | 68.17 | 11.7 | -6.50 [ -10.56, -2.44] | 4.27 |
| Kabisch et al., 2021 | 66 | 83.68 | 8.89 | 33 | 82.91 | 10.37 | 0.77 [ -3.16, 4.70] | 4.30 |
| Kabisch et al., 2021 | 66 | 80.79 | 8.65 | 33 | 78.55 | 8.6 | 2.24 [ -1.37, 5.85] | 4.37 |
| Lee et al., 2009 | 12 | 68.4 | 12.1 | 12 | 74.1 | 16.9 | -5.70 [ -17.46, 6.06] | 2.35 |
| Lee et al., 2015 | 11 | 58.5 | 7.3 | 11 | 60 | 6.96 | -1.50 [ -7.46, 4.46] | 3.79 |
| Li et al., 2021 | 24 | 82.56 | 10.21 | 24 | 78.91 | 10.28 | 3.65 [ -2.15, 9.45] | 3.83 |
| Park et al., 2010 | 268 | 59.89 | 10.71 | 268 | 60.79 | 10.13 | -0.90 [ -2.66, 0.86] | 4.68 |
| Park et al., 2010 | 75 | 66.41 | 8.31 | 75 | 67.86 | 8.84 | -1.45 [ -4.20, 1.30] | 4.54 |
| Pratiwi et al., 2019 | 24 | 68.53 | 12.8 | 24 | 78.32 | 9.84 | -9.79 [ -16.25, -3.33] | 3.65 |
| Song et al., 2019 | 65 | 54.9 | 8.87 | 65 | 55.5 | 8.06 | -0.60 [ -3.51, 2.31] | 4.51 |
| Tsunetsugu et al., 2007 | 11 | 59.09 | 6.38 | 11 | 63.73 | 5.23 | -4.64 [ -9.52, 0.24] | 4.07 |
| Tsunetsugu et al., 2007 | 9 | 62.77 | 5.8 | 9 | 64.6 | 8.32 | -1.83 [ -8.46, 4.80] | 3.61 |
| Heterogeneity: $\tau^2 = 4.19$, $I^2 = 52.77\%$, $H^2 = 2.12$ | | | | | | | -1.60 [ -3.33, 0.12] | |
| Test of $\theta_i = \theta_j$: Q(11) = 23.29, p = 0.02 | | | | | | | | |
| **≥ 20 minutes** | | | | | | | | |
| Aziz et al., 2021 | 90 | 70.3 | 14.89 | 90 | 73 | 10.06 | -2.70 [ -6.41, 1.01] | 4.34 |
| Bang et al., 2016 | 18 | 76.94 | 11.02 | 27 | 72.44 | 10.89 | 4.50 [ -2.03, 11.03] | 3.63 |
| Gong et al., 2017 | 19 | 67.89 | 9.5 | 20 | 60.33 | 6.66 | 7.56 [ 2.43, 12.69] | 4.00 |
| Gong et al., 2017 | 19 | 68 | 7.94 | 20 | 61.08 | 6.24 | 6.92 [ 2.45, 11.39] | 4.17 |
| Janeczko et al., 2020 | 30 | 69.84 | 9.73 | 45 | 75.95 | 11.03 | -6.11 [ -10.98, -1.24] | 4.07 |
| Kanelli et al., 2021 | 24 | 71.5 | 10.29 | 24 | 73.5 | 8.33 | -2.00 [ -7.30, 3.30] | 3.96 |
| Lee and Lee, 2014 | 43 | 66.58 | 13.7 | 19 | 83.11 | 8.94 | -16.53 [ -23.26, -9.80] | 3.58 |
| Lei et al., 2020 | 9 | 84.81 | 4.13 | 9 | 85.58 | 3.17 | -0.77 [ -4.17, 2.63] | 4.41 |
| Lyu et al., 2019 | 45 | 63.64 | 9.66 | 15 | 61.9 | 9.06 | 1.74 [ -3.82, 7.30] | 3.89 |
| Mao et al., 2012 | 12 | 80.84 | 6.97 | 12 | 87.19 | 7.28 | -6.35 [ -12.05, -0.65] | 3.85 |
| Pratiwi et al., 2020 | 36 | 72.83 | 12.7 | 36 | 78.23 | 13.01 | -5.40 [ -11.34, 0.54] | 3.79 |
| Stigsdotter et al., 2017 | 51 | 70.2 | 7.98 | 51 | 72.5 | 8.48 | -2.30 [ -5.50, 0.90] | 4.45 |
| Zheng et al., 2017 | 95 | 62 | 14 | 95 | 96 | 24 | -34.00 [ -39.59, -28.41] | 3.88 |
| Heterogeneity: $\tau^2 = 83.03$, $I^2 = 93.42\%$, $H^2 = 15.20$ | | | | | | | -4.18 [ -9.33, 0.98] | |
| Test of $\theta_i = \theta_j$: Q(12) = 182.37, p = 0.00 | | | | | | | | |
| **Overall** | | | | | | | -3.07 [ -5.59, -0.54] | |
| Heterogeneity: $\tau^2 = 34.72$, $I^2 = 88.59\%$, $H^2 = 8.76$ | | | | | | | | |
| Test of $\theta_i = \theta_j$: Q(24) = 210.26, p = 0.00 | | | | | | | | |
| Test of group differences: $Q_b(1) = 0.86$, p = 0.35 | | | | | | | | |

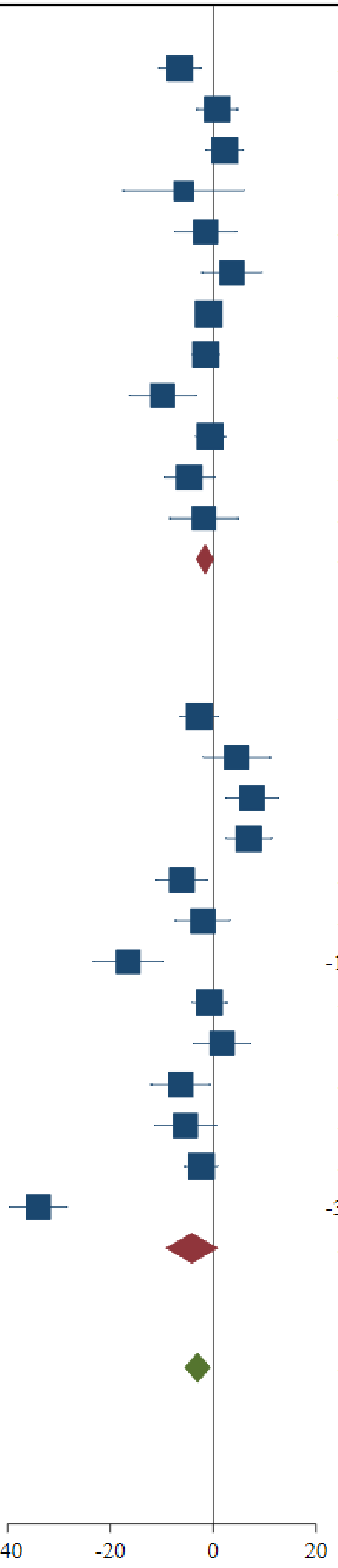


Random-effects DerSimonian–Laird model

**Figure S13. Study design-based subgroup analysis of the effects of forest therapy and urban control on salivary cortisol concentration**

| Subgroup | Treatment N | Treatment Mean | Treatment SD | Control N | Control Mean | Control SD | Mean difference with 95% CI | Weight (%) |
|---|---|---|---|---|---|---|---|---|
| **RCT** | | | | | | | | |
| Gidlow et al., 2016 | 37 | .14 | .08 | 37 | .14 | .06 | 0.00 [ -0.03, 0.03] | 7.75 |
| Kobayashi et al., 2017 | 348 | .25 | .1 | 348 | .29 | .12 | -0.04 [ -0.06, -0.02] | 8.34 |
| Komori et al., 2017 | 22 | .14 | .09 | 22 | .18 | .09 | -0.04 [ -0.09, 0.01] | 6.66 |
| Lee et al., 2009 | 12 | .24 | .04 | 12 | .27 | .07 | -0.03 [ -0.08, 0.02] | 7.08 |
| Lee et al., 2011 | 12 | .35 | .14 | 12 | .41 | .14 | -0.06 [ -0.17, 0.05] | 3.78 |
| Lee et al., 2015 | 11 | .22 | .07 | 11 | .29 | .12 | -0.07 [ -0.15, 0.01] | 5.09 |
| Park et al., 2007 | 9 | .33 | .1 | 9 | .69 | .06 | -0.36 [ -0.44, -0.28] | 5.40 |
| Park et al., 2007 | 12 | .6 | .09 | 12 | .55 | .09 | 0.05 [ -0.02, 0.12] | 5.62 |
| Park et al., 2008 | 10 | .27 | .08 | 10 | .32 | .1 | -0.05 [ -0.13, 0.03] | 5.23 |
| Park et al., 2010 | 260 | .3 | .14 | 260 | .34 | .17 | -0.04 [ -0.07, -0.01] | 7.99 |
| Park et al., 2010 | 74 | .3 | .11 | 74 | .36 | .14 | -0.06 [ -0.10, -0.02] | 7.34 |
| Tsunetsugu et al., 2007 | 11 | .26 | .03 | 11 | .36 | .11 | -0.10 [ -0.17, -0.03] | 5.87 |
| Tsunetsugu et al., 2007 | 9 | .37 | .1 | 9 | .49 | .15 | -0.12 [ -0.24, -0.00] | 3.57 |
| Heterogeneity: $\tau^2 = 0.00$, $I^2 = 86.02\%$, $H^2 = 7.15$ | | | | | | | -0.06 [ -0.10, -0.03] | |
| Test of $\theta_i = \theta_j$: Q(12) = 85.82, p = 0.00 | | | | | | | | |
| **Non-RCT** | | | | | | | | |
| Juong et al., 2020 | 22 | .26 | .09 | 22 | .3 | .09 | -0.04 [ -0.09, 0.01] | 6.66 |
| Kanelli et al., 2021 | 24 | .1 | .05 | 24 | .2 | .1 | -0.10 [ -0.14, -0.06] | 7.12 |
| Toda et al., 2013 | 20 | .35 | .08 | 20 | .43 | .1 | -0.08 [ -0.14, -0.02] | 6.50 |
| Heterogeneity: $\tau^2 = 0.00$, $I^2 = 30.66\%$, $H^2 = 1.44$ | | | | | | | -0.08 [ -0.11, -0.04] | |
| Test of $\theta_i = \theta_j$: Q(2) = 2.88, p = 0.24 | | | | | | | | |
| **Overall** | | | | | | | -0.07 [ -0.10, -0.04] | |
| Heterogeneity: $\tau^2 = 0.00$, $I^2 = 83.85\%$, $H^2 = 6.19$ | | | | | | | | |
| Test of $\theta_i = \theta_j$: Q(15) = 92.91, p = 0.00 | | | | | | | | |
| Test of group differences: $Q_b(1) = 0.19$, p = 0.67 | | | | | | | | |

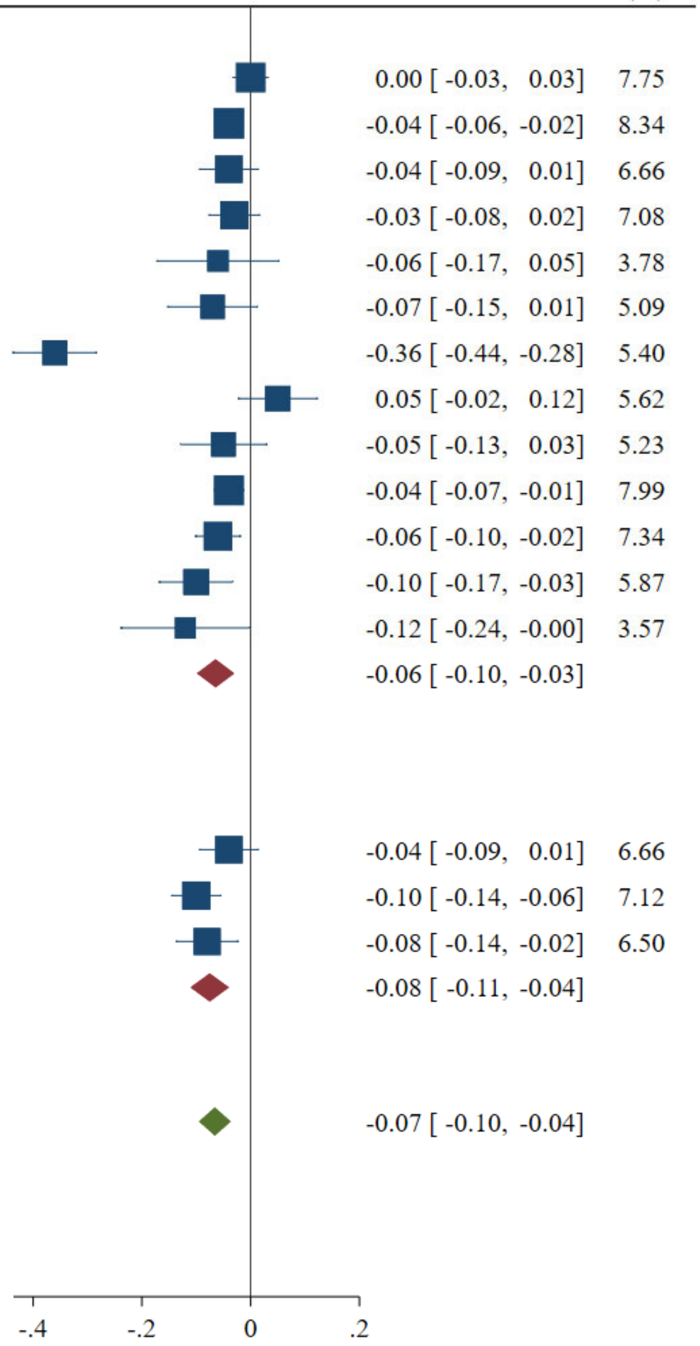


Random-effects DerSimonian–Laird model

**Figure S14. Session-based subgroup analysis of the effects of forest therapy and urban control on salivary cortisol concentration**

| Subgroup | Treatment N | Treatment Mean | Treatment SD | Control N | Control Mean | Control SD | Mean difference with 95% CI | Weight (%) |
|---|---|---|---|---|---|---|---|---|
| **Seated viewing** | | | | | | | | |
| Kobayashi et al., 2017 | 348 | .25 | .1 | 348 | .29 | .12 | -0.04 [ -0.06, -0.02] | 8.34 |
| Lee et al., 2009 | 12 | .24 | .04 | 12 | .27 | .07 | -0.03 [ -0.08, 0.02] | 7.08 |
| Lee et al., 2011 | 12 | .35 | .14 | 12 | .41 | .14 | -0.06 [ -0.17, 0.05] | 3.78 |
| Lee et al., 2015 | 11 | .22 | .07 | 11 | .29 | .12 | -0.07 [ -0.15, 0.01] | 5.09 |
| Park et al., 2007 | 9 | .33 | .1 | 9 | .69 | .06 | -0.36 [ -0.44, -0.28] | 5.40 |
| Park et al., 2008 | 10 | .27 | .08 | 10 | .32 | .1 | -0.05 [ -0.13, 0.03] | 5.23 |
| Park et al., 2010 | 260 | .3 | .14 | 260 | .34 | .17 | -0.04 [ -0.07, -0.01] | 7.99 |
| Tsunetsugu et al., 2007 | 11 | .26 | .03 | 11 | .36 | .11 | -0.10 [ -0.17, -0.03] | 5.87 |
| Heterogeneity: $\tau^2 = 0.00$, $I^2 = 89.80\%$, $H^2 = 9.81$ | | | | | | | -0.09 [ -0.14, -0.04] | |
| Test of $\theta_i = \theta_j$: $Q(7) = 68.64$, $p = 0.00$ | | | | | | | | |
| **Walking or multi-session program** | | | | | | | | |
| Gidlow et al., 2016 | 37 | .14 | .08 | 37 | .14 | .06 | 0.00 [ -0.03, 0.03] | 7.75 |
| Juong et al., 2020 | 22 | .26 | .09 | 22 | .3 | .09 | -0.04 [ -0.09, 0.01] | 6.66 |
| Kanelli et al., 2021 | 24 | .1 | .05 | 24 | .2 | .1 | -0.10 [ -0.14, -0.06] | 7.12 |
| Komori et al., 2017 | 22 | .14 | .09 | 22 | .18 | .09 | -0.04 [ -0.09, 0.01] | 6.66 |
| Park et al., 2007 | 12 | .6 | .09 | 12 | .55 | .09 | 0.05 [ -0.02, 0.12] | 5.62 |
| Park et al., 2010 | 74 | .3 | .11 | 74 | .36 | .14 | -0.06 [ -0.10, -0.02] | 7.34 |
| Toda et al., 2013 | 20 | .35 | .08 | 20 | .43 | .1 | -0.08 [ -0.14, -0.02] | 6.50 |
| Tsunetsugu et al., 2007 | 9 | .37 | .1 | 9 | .49 | .15 | -0.12 [ -0.24, -0.00] | 3.57 |
| Heterogeneity: $\tau^2 = 0.00$, $I^2 = 70.17\%$, $H^2 = 3.35$ | | | | | | | -0.05 [ -0.08, -0.01] | |
| Test of $\theta_i = \theta_j$: $Q(7) = 23.46$, $p = 0.00$ | | | | | | | | |
| **Overall** | | | | | | | -0.07 [ -0.10, -0.04] | |
| Heterogeneity: $\tau^2 = 0.00$, $I^2 = 83.85\%$, $H^2 = 6.19$ | | | | | | | | |
| Test of $\theta_i = \theta_j$: $Q(15) = 92.91$, $p = 0.00$ | | | | | | | | |
| Test of group differences: $Q_b(1) = 2.02$, $p = 0.16$ | | | | | | | | |

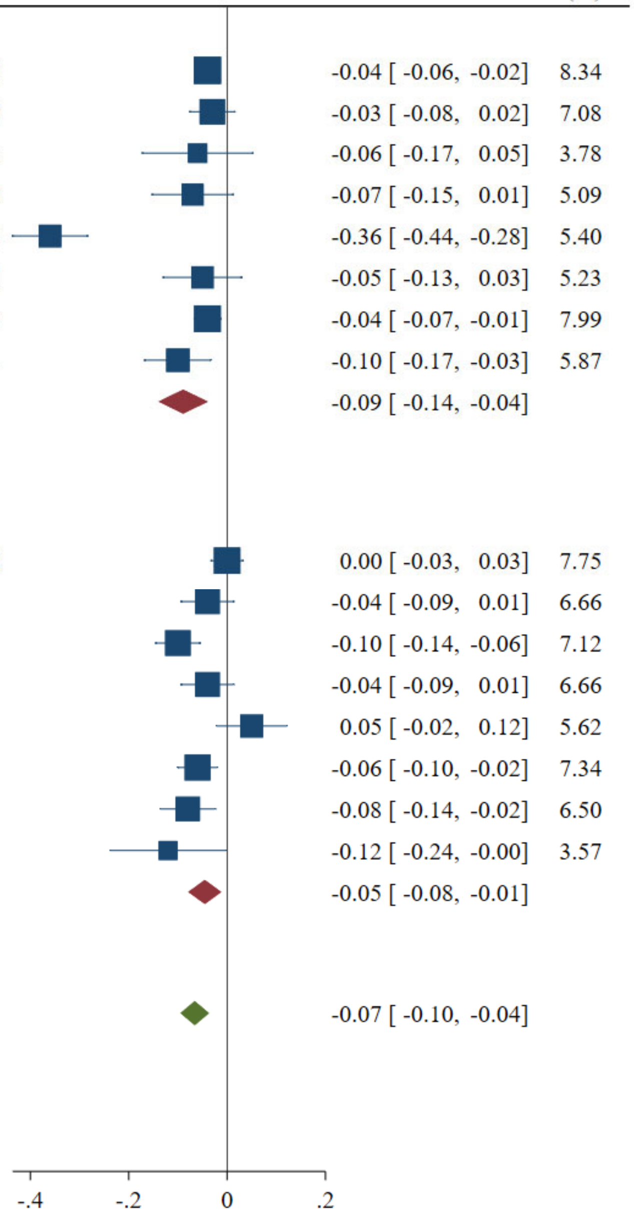


Random-effects DerSimonian–Laird model

**Figure S15. Duration-based subgroup analysis of the effects of forest therapy and urban control on salivary cortisol concentration**

| Subgroup | Treatment N | Treatment Mean | Treatment SD | Control N | Control Mean | Control SD | Mean difference with 95% CI | Weight (%) |
|---|---|---|---|---|---|---|---|---|
| **< 20 minutes** | | | | | | | | |
| Juong et al., 2020 | 22 | .26 | .09 | 22 | .3 | .09 | -0.04 [ -0.09, 0.01] | 6.66 |
| Kobayashi et al., 2017 | 348 | .25 | .1 | 348 | .29 | .12 | -0.04 [ -0.06, -0.02] | 8.34 |
| Lee et al., 2009 | 12 | .24 | .04 | 12 | .27 | .07 | -0.03 [ -0.08, 0.02] | 7.08 |
| Lee et al., 2011 | 12 | .35 | .14 | 12 | .41 | .14 | -0.06 [ -0.17, 0.05] | 3.78 |
| Lee et al., 2015 | 11 | .22 | .07 | 11 | .29 | .12 | -0.07 [ -0.15, 0.01] | 5.09 |
| Park et al., 2008 | 10 | .27 | .08 | 10 | .32 | .1 | -0.05 [ -0.13, 0.03] | 5.23 |
| Park et al., 2010 | 260 | .3 | .14 | 260 | .34 | .17 | -0.04 [ -0.07, -0.01] | 7.99 |
| Park et al., 2010 | 74 | .3 | .11 | 74 | .36 | .14 | -0.06 [ -0.10, -0.02] | 7.34 |
| Tsunetsugu et al., 2007 | 11 | .26 | .03 | 11 | .36 | .11 | -0.10 [ -0.17, -0.03] | 5.87 |
| Tsunetsugu et al., 2007 | 9 | .37 | .1 | 9 | .49 | .15 | -0.12 [ -0.24, -0.00] | 3.57 |
| Heterogeneity: $\tau^2 = 0.00$, $I^2 = 0.00\%$, $H^2 = 1.00$ | | | | | | | -0.04 [ -0.06, -0.03] | |
| Test of $\theta_i = \theta_j$: Q(9) = 6.02, p = 0.74 | | | | | | | | |
| **≥ 20 minutes** | | | | | | | | |
| Gidlow et al., 2016 | 37 | .14 | .08 | 37 | .14 | .06 | 0.00 [ -0.03, 0.03] | 7.75 |
| Kanelli et al., 2021 | 24 | .1 | .05 | 24 | .2 | .1 | -0.10 [ -0.14, -0.06] | 7.12 |
| Komori et al., 2017 | 22 | .14 | .09 | 22 | .18 | .09 | -0.04 [ -0.09, 0.01] | 6.66 |
| Park et al., 2007 | 9 | .33 | .1 | 9 | .69 | .06 | -0.36 [ -0.44, -0.28] | 5.40 |
| Park et al., 2007 | 12 | .6 | .09 | 12 | .55 | .09 | 0.05 [ -0.02, 0.12] | 5.62 |
| Toda et al., 2013 | 20 | .35 | .08 | 20 | .43 | .1 | -0.08 [ -0.14, -0.02] | 6.50 |
| Heterogeneity: $\tau^2 = 0.01$, $I^2 = 94.18\%$, $H^2 = 17.17$ | | | | | | | -0.09 [ -0.17, 0.00] | |
| Test of $\theta_i = \theta_j$: Q(5) = 85.85, p = 0.00 | | | | | | | | |
| **Overall** | | | | | | | -0.07 [ -0.10, -0.04] | |
| Heterogeneity: $\tau^2 = 0.00$, $I^2 = 83.85\%$, $H^2 = 6.19$ | | | | | | | | |
| Test of $\theta_i = \theta_j$: Q(15) = 92.91, p = 0.00 | | | | | | | | |
| Test of group differences: $Q_b(1) = 0.85$, p = 0.36 | | | | | | | | |

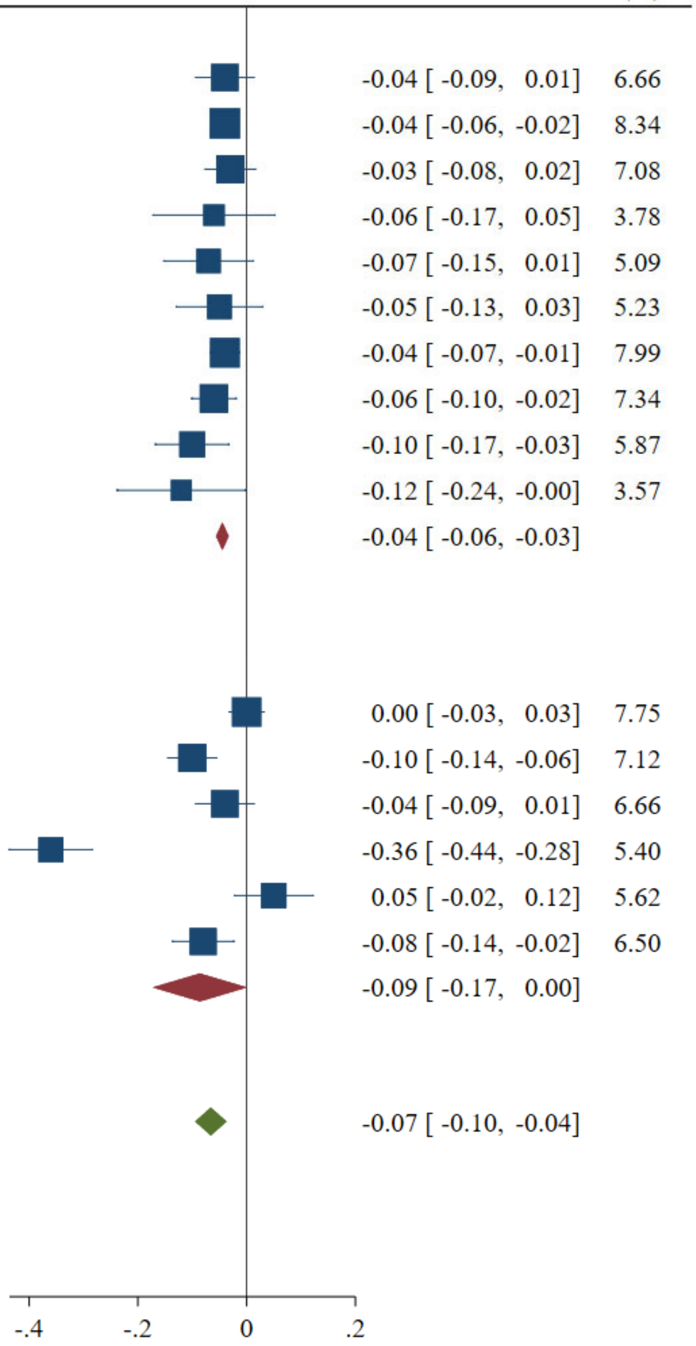


Random-effects DerSimonian–Laird model
